# Insights into Genetic Characteristics and Virological Features of Endemic Avian Influenza A (H9N2) Viruses in Egypt from 2017–2021

**DOI:** 10.3390/v14071484

**Published:** 2022-07-06

**Authors:** Mohamed El Sayes, Ahmed Kandeil, Yassmin Moatasim, Ahmed El Taweel, Adam Rubrum, Omnia Kutkat, Mina Nabil Kamel, Rebecca Badra, Ahmed B. Barakat, Pamela P. McKenzie, Rabeh El-Shesheny, Richard J. Webby, Ghazi Kayali, Mohamed Ahmed Ali

**Affiliations:** 1 Center of Scientific Excellence for Influenza Viruses, National Research Centre, Giza 12622, Egypt; mohameddiaaelsayes@outlook.com (M.E.S.); yasmin.moatasim@human-link.org (Y.M.); ahmed.nageh@human-link.org (A.E.T.); omnia.abdelaziz@human-link.org (O.K.); mina@human-link.org (M.N.K.); rabeh.elshesheny@human-link.org (R.E.-S.); mohamedahmedali2004@yahoo.com (M.A.A.); 2 Department of Infectious Diseases, St. Jude Children’s Research Hospital, Memphis, TN 38105, USA; adam.rubrum@stjude.org (A.R.); pamela.mckenzie@stjude.org (P.P.M.); 3 Human Link, Dubai 3O-01-BA380, United Arab Emirates; rebecca@human-link.org; 4 Department of Microbiology, Faculty of Science, Ain Shams University, Cairo 11566, Egypt; dr.barakat51@hotmail.com

**Keywords:** avian influenza, H9N2, Egypt, surface glycoproteins, genetic evolution, replication rate, reassortant

## Abstract

From 2010 to 2013, genotype I avian influenza A(H9N2) viruses of the G1-lineage were isolated from several poultry species in Egypt. In 2014, novel reassortant H9N2 viruses were detected in pigeons designated as genotype II. To monitor the subsequent genetic evolution of Egyptian A(H9N2) viruses, we characterized the full genomes of 173 viruses isolated through active surveillance from 2017 to 2022. In addition, we compared the virological characteristics and pathogenicity of representative viruses. Phylogenetic analysis of the HA indicated that all studied sequences from 2017–2021 were grouped into G1-like H9N2 viruses previously detected in Egypt. Phylogenetic analysis indicated that the Egyptian A(H9N2) viruses had undergone further reassortment, inheriting four genes (PB2, PB1, PA, NS) from genotype II, with their remaining segments deriving from genotype I viruses (these viruses designated as genotype III). Studying the virological features of the two most dominant genotypes (I and III) of Egyptian H9N2 viruses in vitro and in vivo indicated that both replicated well in mammalian cells, but did not show any clinical signs in chickens, ducks, and mice. Monitoring avian influenza viruses through surveillance programs and understanding the genetic and antigenic characteristics of circulating H9N2 viruses are essential for risk assessment and influenza pandemic preparedness.

## 1. Introduction

The avian influenza (AI) H9N2 virus, a subtype of avian influenza A that belongs to the family Orthomyxoviridae, was first isolated from turkeys in the United States in 1966 [1]. This low pathogenic virus was phylogenetically split into two major groups, American and Eurasian, based on genetic differences in the hemagglutinin (HA) segment [2]. The Eurasian group of H9N2 viruses was divided into three main lineages, the Y280 lineage, the Korean lineage, and the G1-like lineage, based on phylogenetic and antigenic analyses [3,4,5]. The G1-like lineage is the most prevalent lineage and is subsequently divided into four groups: A, B, C, and D [6,7]. The AI H9N2 virus became endemic among poultry in many Middle Eastern countries, especially Saudi Arabia, Israel, Jordan, and Egypt [8,9,10]. Based on the HA sequence, the Egyptian H9N2 viruses belonged to group B of G1-like lineage [11,12]. AI H9N2 viruses were then isolated from commercial broilers, broiler breeders, and layer farms [13,14,15,16]. In Egypt, AI H9N2 viruses have been isolated from several poultry populations, including turkeys, chickens, pigeons, and quails [17,18,19]. H9N2 viruses can infect layers of chickens, leading to a drop in egg production [10,13]. On the other hand, most infected quail and broiler flocks with the H9N2 virus appear healthy, with no clinical signs [12]. In general, infection with H9N2 viruses in chickens is more prevalent than in ducks [20]. In 2014, novel reassortant H9N2 viruses were detected in pigeons in Egypt. These viruses inherited three genes (HA, NA, and M) from the endemic H9N2 viruses in Egypt and five genes (PB2, PB1, PA, NP, and NS) from Eurasian AI viruses (AIVs) circulating in wild birds [21]. In addition to different species of domestic poultry, Egyptian fruit bats and wild migratory birds were found to be infected with H9N2 viruses in Egypt [22,23]. Additionally, several H9N2 infections in humans have been reported. The majority of infections were likely due to direct contact with poultry infected with H9N2 viruses. The symptoms of human H9N2 infections were mild, and only one death has been reported. The Egyptian Ministry of Health reported three laboratory-confirmed human infections of H9N2 [24]. To monitor the genetic evolution of Egyptian H9N2 viruses and determine the possibility of a novel reassortment, the characteristics of surface glycoproteins of 173 H9N2 viruses isolated through the active surveillance of AI in Egypt between 2017 and 2021 were studied. Whole genome sequences of 173 H9N2 viruses isolated from different hosts were phylogenetically analyzed. In addition, we compared the virological characteristics and pathogenicity of the most dominant form of the H9N2 virus detected in poultry in Egypt and the first parent detected during the first introduction wave in 2011.

## 2. Materials and Methods

### 2.1. Genetic Analysis

#### 2.1.1. Viruses

During active surveillance of AIVs in six Egyptian governorates, 173 H9N2 viruses were isolated from poultry from March 2017 to February 2021 (55 H9N2 isolates from Assiut, 19 from Dakahliya, 11 from Fayoum, 36 from Menia, 6 from Kalyobiya, and 46 from Sharqeia governorates). Isolates were collected from healthy, sick, and dead poultry. The details of isolation area, health status of the host, date of isolation, and site of sampling (commercial farm, market, house) of these isolates are given in Table S1. The 173 isolates from different hosts (1 isolate from duck, 7 isolates from pigeons, and 165 isolates from chickens) were inoculated in 10-day-old specific-pathogen-free embryonated chicken eggs, incubated at 37 °C for 48 h and chilled at 4 °C for 4 h. Allantoic fluids were harvested, cleared by centrifugation, aliquoted, and transferred to a –80 °C freezer for long-term storage. Two isolates were obtained from broiler chickens vaccinated with the inactivated H5N1 vaccine, seven were isolated from broiler chickens vaccinated with the inactivated H9N2 vaccine, four isolates were from broilers chickens vaccinated with inactivated H5N1 and H9N2 vaccine, and three isolates were from broiler chickens vaccinated with the H9N2 and Newcastle Disease Virus (NDV) vaccines.

#### 2.1.2. Amplification of Full Genome and Sequencing

The H9N2 isolates propagated were used for RNA extraction using the QIAamp Viral RNA Mini Kit (Qiagen, Hilden, Germany) according to the manufacturer’s instructions. Extracted RNAs were used for cDNA synthesis using uni12 primer [25] and the Superscript III Reverse Transcriptase kit (Invitrogen, Carlsbad, CA, USA). Then, Phusion™ high-fidelity DNA polymerase (New England Biolabs, Ipswich, MA, USA) and Uni12/13 primers were used for multiplex PCR of all eight gene segments, and PCR products were purified. The staff of the Hartwell Center at St. Jude Children’s Research Hospital prepared the DNA libraries, which were then pooled and sequenced using 150 bp paired-end sequencing using the Illumina MiSeq personal genome sequencer. The sequencing reads were analyzed using CLC Genomics Workbench, version 20 (CLC Bio, Qiagen, Hilden, Germany). Sequences were deposited in GenBank under the accession numbers listed in Table S2.

#### 2.1.3. Sequence Analysis and Phylogenetic Tree Construction

The assembled sequences underwent NCBI BLAST analysis. BioEdit 7.0 was used for multiple sequence alignment [26]. The nucleotide and amino acid homologies were further assessed by the ClustalW method MegAlign (DNASTAR). MEGA 7 was used for phylogenetic tree construction by applying the neighbor-joining method with Kimura’s two-parameter distance model and 1000 bootstrap replicates [27]. The trees included all Egyptian H9N2 virus sequences, major ancestral H9N2 strains, and other influenza virus subtypes with closely related H9N2 genes, as shown by BLASTN analysis. Sequences were obtained from the Global Initiative on Sharing All Influenza Data (GISAID) and GenBank. The BioEdit program version 7.0 was used for genomic signature analysis.

### 2.2. Virological Characteristics of Egyptian H9N2 Viruses

#### 2.2.1. Cells

Madin Darby canine kidney (MDCK) cells and Madin Darby canine kidney (MDCK-SIAT) cells were obtained from St. Jude Children’s Research Hospital, USA and cultured in Dulbecco’s Modified Eagle’s Medium (DMEM) (BioWhittaker, Lonza, Cologne, Germany) supplemented with 5% inactivated fetal bovine serum (FBS; Gibco BRL Life Technologies, Grand Island, NY, USA) and 1% antibiotic-antimycotic mixture (BioWhittaker, Lonza, Cologne, Germany) and grown at 37 °C and 5% CO_2_.

#### 2.2.2. Viruses

Based on genetic analysis, we selected representative H9N2 viruses of the most dominant genotypes. The plaque-purified A/chicken/Egypt/S4456B/2011 (H9N2; abbreviated as S4456B; genotype I) and A/chicken/Egypt/A17358/2019 (H9N2; abbreviated as A17358, genotype III) viruses were propagated in allantoic cavities of 11-day-old specific pathogen-free embryonated chicken eggs (SPF-ECEs) for 48 h. The harvests were aliquoted and stored at −80 °C until use. Each virus was titrated using the 50% tissue culture infectious dose assay (TCID50/mL) in MDCK cells and the 50% egg infectious dose assay (EID50/mL) in SPF-ECEs, and titers were calculated using the Reed and Muench method [28].

#### 2.2.3. Growth Kinetics of H9N2 Viruses in Mammalian Cells

Growth kinetics of the two genotypes of Egyptian H9N2 viruses were compared in MDCK and MDCK-SIAT cells. Each virus was inoculated into the three types of cell monolayers, with a multiplicity of infection (MOI) of 0.01. The supernatants of infected cells were collected at specific time points hours post-infection (hpi) and kept at −80 °C. The titer of each collected sample was determined using the HA and TaqMan Real-Time PCR assays.

#### 2.2.4. Replication Rate of H9N2 Viruses in SPF-ECEs

The growth properties of the two viruses were compared in SPF-ECEs (Koum Oshiem SPF Chicken Farm, Fayoum, Egypt) by inoculating 10^6^ EID_50_ of each virus into five SPF eggs. The allantoic fluids were harvested at specific time points and titrated using the HA assay and EID50/100 µL.

#### 2.2.5. Animal Experiments

Animal experiments were approved by the Medical Research Ethics Committee of the National Research Centre (Ethical permission code: 18040 in April 2018).

#### 2.2.6. Pathogenicity in Chickens and Ducks

To determine the pathogenicity of the Egyptian H9N2 viruses in chickens and ducks, 4-week-old SPF White Leghorn chickens and 3-week-old Pekin ducks were divided into three groups. The first group was infected with 10^7^ EID_50_ in 100 µL of the S4456B virus, and the second was infected with 10^7^ EID_50_ in 100 µL of the A17358 virus. The third was the control group. Birds were infected through natural routes (intranasal, intraocular, or intratracheal). Oral and cloacal swabs were collected from three birds per group at 3, 6, and 10 days post-infection (PI), and different organs were collected from three animals per group at 3 and 6 days PI. To determine viral titers in organs, 0.1 gm of each organ were homogenized in 0.9 mL Phosphate Buffer Saline (PBS) with a Qiagen Tissue Lyser II (Qiagen, Hilden, Germany). Organ homogenates were centrifuged at 2000× *g* for 5 min. Swabs and homogenates of organs underwent virus titration by EID_50_. Sera samples were collected from each experimental group two weeks PI and analyzed by the hemagglutination inhibition (HI) assay to test for seroconversion.

#### 2.2.7. Pathogenicity in Mice

Three groups of 11 C57BL/six mice (6 to 8 weeks old) were anesthetized with isoflurane and intranasally inoculated with 10^7^ EID_50_ in 20 µL of the S4456B virus, A17358, or PBS. Five mice per group were monitored for 14 days PI for body weight loss and mortality. Mortality was recorded as actual death or loss of ≥25% of body weight (the threshold at which animals were euthanized). Three mice per group were euthanized at 3 and 6 days PI. The lungs, spleen, intestine, liver, brain, and kidneys were collected. To determine viral titers, 0.1 gm of each organ were homogenized in 0.9 mL PBS using Qiagen Tissue Lyser II (Qiagen, Hilden, Germany). Organ homogenates were centrifuged at 2000× *g* for 5 min, and the virus titer was determined in the supernatants by TaqMan Real-Time PCR. Sera samples were collected from each experimental group on day 14 PI and were analyzed by the HI assay to test for seroconversion.

### 2.3. Vaccine Evaluation

#### 2.3.1. Vaccine Preparation

The A17358 virus was inactivated by adding 0.1% formalin overnight. The complete inactivation process for each antigen used in this study was verified by inoculating the inactivated antigens into 11-day-old SPF ECEs. Inactivated antigens were mixed with adjuvant Montanide™ ISA 71 VG (Seppic Inc., Puteaux, France) as a water-in-oil (W/O) emulsion at the ratio recommended in the manufacturer’s technical manual (30 antigen/70 adjuvant W/W), followed by homogenization for 3 min on ice using a mixer homogenizer. To test the safety of the prepared vaccine, five chickens (3-week-old) were inoculated intramuscularly by a double dose (1 mL) of the vaccine and then observed for the presence of clinical signs or local lesions at the site of vaccination for two weeks [29].

#### 2.3.2. Immunization of SPF Chickens

Seventy 2-week-old SPF chickens were purchased from Koum Oshiem SPF Chicken Farm, Fayoum, Egypt and divided into two groups: group 1 included 26 chickens immunized with the A17358/H9N2 vaccine, and group 2 included 44 unimmunized chickens as the control group. Further, after seven weeks, they were infected with the A17358 and S4456B viruses in order to evaluate the differences in pathogenicity between the two H9N2 viruses. Chickens were vaccinated intramuscularly in the leg with 0.5 mL of the required vaccine. Serum was collected weekly from all chickens to determine serological conversion.

Specific antibodies were determined by the HI assay and virus microneutralization test (VMN) [30]. Homologous A17358/H9N2 and heterologous S4456B/H9N2 viruses were used for titrating specific antibodies in both HI and VMN assays. Sera collected were analyzed using the HI and VMN assays against the two forms of H9N2 viruses. Briefly, a standardized quantity of HA antigen (4 HA units) is mixed with serially diluted serum samples, and red blood cells (RBCs) are added to detect the specific binding of the antibody to the HA molecule. The presence of specific anti-HA antibodies will inhibit agglutination, which would have otherwise occurred between the virus and RBCs. Log2 serially diluted sera were incubated at a 50 TCID50 dilution of the virus for 1 hr prior to the infection of MDCK cells. Then, the virus–serum mix was removed, 200 µL of infection media were added to the cells, and cells were incubated for three days. Inhibition was determined by the HA assay.

### 2.4. Statistical Analysis

GraphPad Prism V5 (GraphPad Inc., La Jolla, CA, USA) was used for statistical analysis. Statistical analysis was performed using the one-way ANOVA test, followed by the Bonferroni post-hoc test. Data were represented as mean ± SD. *p* values of ≤0.05 were considered statistically significant.

## 3. Results

### 3.1. Genetic Analysis of Internal Proteins

#### 3.1.1. PB2

**Figure 1:**
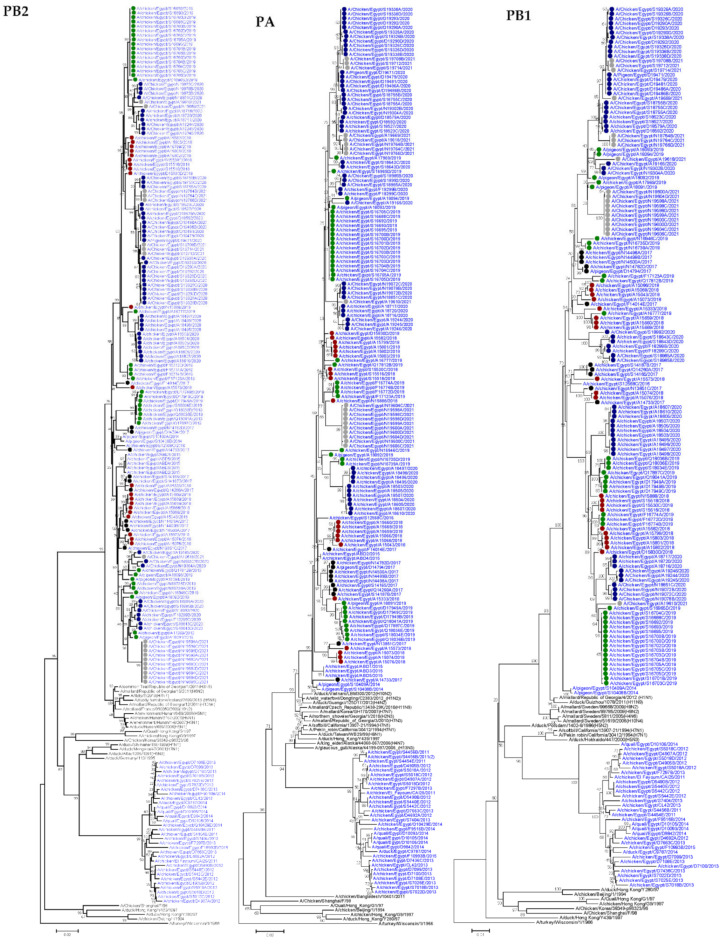

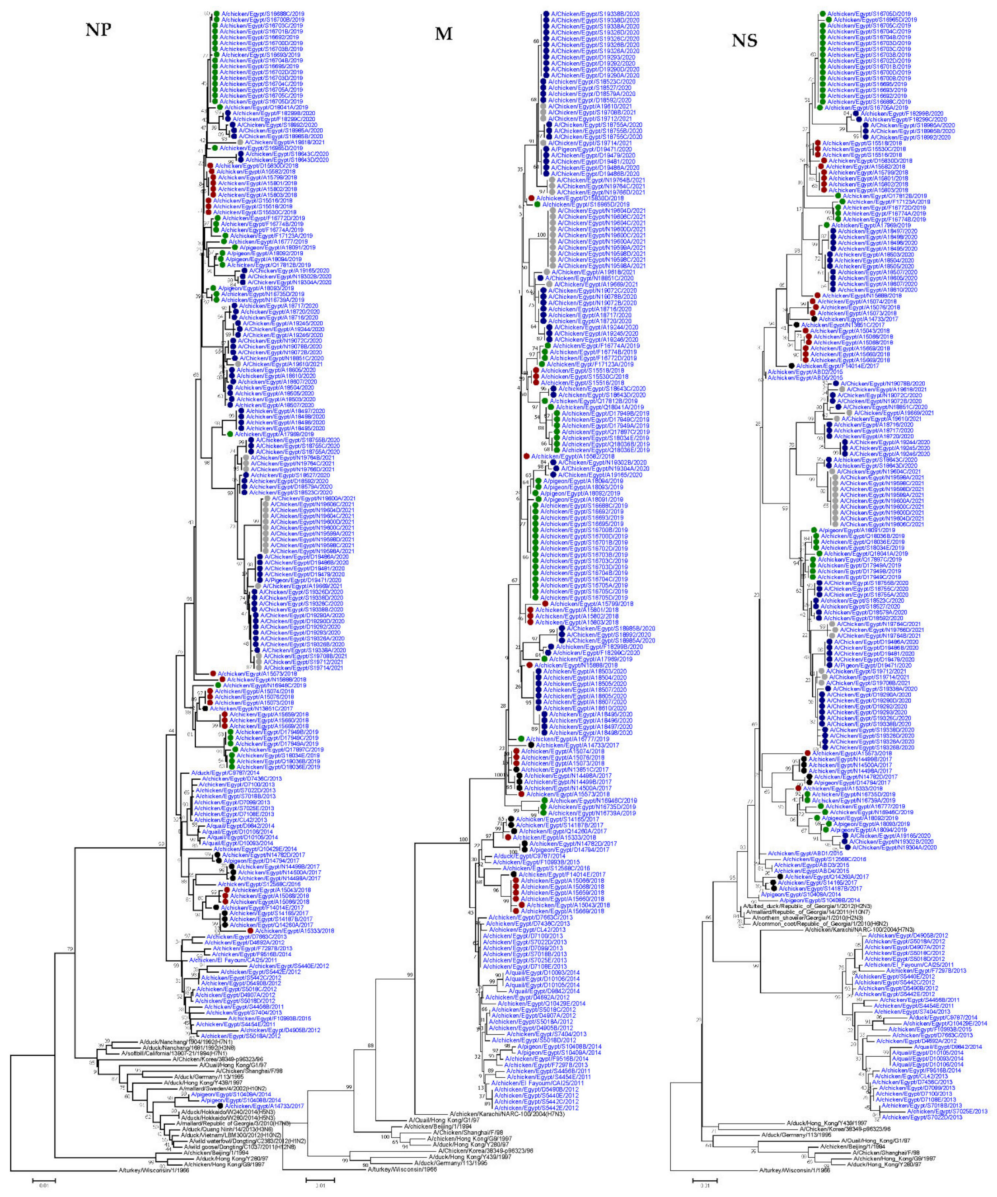
Neighbor joining phylogenetic trees of the six internal segments of the H9N2 viruses detected in Egypt. All Egyptian H9N2 taxa are colored blue and H9N2 viruses sequenced specifically for this study are labeled with colored circles (2017 black, 2018 red, 2019 green, 2020 blue, and 2021 gray). The phylogenetic analysis was performed using MEGA7, and evolutionary distances were computed using the Kimura 2-parameter method.

The PB2 genes of the 173 Egyptian H9N2 isolates reported in the current study showed a higher identity to H9N2 viruses isolated previously from pigeons in Egypt in 2014 [20], A/pigeon/Egypt/S10408B/2014 (H9N2), and A/pigeon/Egypt/S10409A/2014 (H9N2), which were closely related to PB2 of an A/common teal/Republic of Georgia/1/2011(H3N8) isolate rather than PB2 of the Egyptian H9N2 viruses circulating from 2010–2014 (Figure 1). Genetic analysis of PB2 segments of the 173 Egyptian H9N2 isolates showed the presence of V at residue 504 in all isolates, which is a virulence marker associated with enhanced activity of the polymerase complex [31]. The substitution of K for R at position 318, which is a mammalian host-specific marker, was observed in all isolates [32] (Table S3). Another mutation associated with mammalian host specificity was detected at position 661 by the presence of T instead of A in one isolate, the A/chicken/Egypt/A15333/2018 (H9N2) virus [33]. Moreover, several mutations associated with mammalian host specificity, including M64T and A199S [34,35,36], were detected in 65 isolates and 1 isolate (A/chicken/Egypt/N19302B/2020), respectively. Other mutations that play an important role in virulence and are associated with virus transmission in mammals included D701N, which was absent in all viruses analyzed, whereas E627K was observed in one virus (A/chicken/Egypt/S18755D/2020) [37,38] (Table 1). Other residues of PB2 were associated with avian preference.

#### 3.1.2. PB1

PB1 genes of the 173 Egyptian H9N2 isolates in the current study showed a higher identity to H9N2 viruses isolated previously from pigeons in Egypt in 2014 [21], A/pigeon/Egypt/S10408B/2014 (H9N2), and A/pigeon/Egypt/S10409A/2014 (H9N2) that were closely related to the PB1 of an A/mallard/Republic of Georgia/4/2012 (H1N1) isolate rather than the PB1 of circulating Egyptian H9N2 viruses from 2010 to 2014 (Figure 1). However, the substitution of N for S at position 375, which is a mammalian host-specific marker [57], was observed in three viruses analyzed: A/chicken/Egypt/A15333/2018, A/chicken/Egypt/D19290B/2020, and A/chicken/Egypt/A19610/2021 (Table S3). The substitution of N for S at position 66 in PB1-F2, which is associated with enhanced viral pathogenesis [58], has not been observed in any of the isolates (Table 1).

#### 3.1.3. PA

Phylogenetic analysis of Egyptian isolates from 2017 to 2021 showed that they clustered with novel reassortant H9N2 viruses isolated in Egypt from pigeons in 2014 (Figure 1) [21]. The PA gene of all Egyptian isolates had several genetic markers associated with virulence, including the presence of V at residue 127 and L at positions 550 and 672 (Table 1). In addition, mammalian host specificity resulting from several substitutions [59], including that from the substitution of R for Q at position 57, was detected in 14 isolates, and amino acid substitution V100A was observed in 13 isolates (Table S3). Furthermore, mammalian preference mutations such as L268I were observed in one virus (A/chicken/Egypt/A19618/2021), and 382D was observed in all isolates except for the PA gene of 18 isolates that possessed E (avian-like marker). The substitution of S for N at position 409 was detected in six viruses analyzed. Other residues of PA were associated with avian host-specific markers.

#### 3.1.4. HA

**Figure 2:**
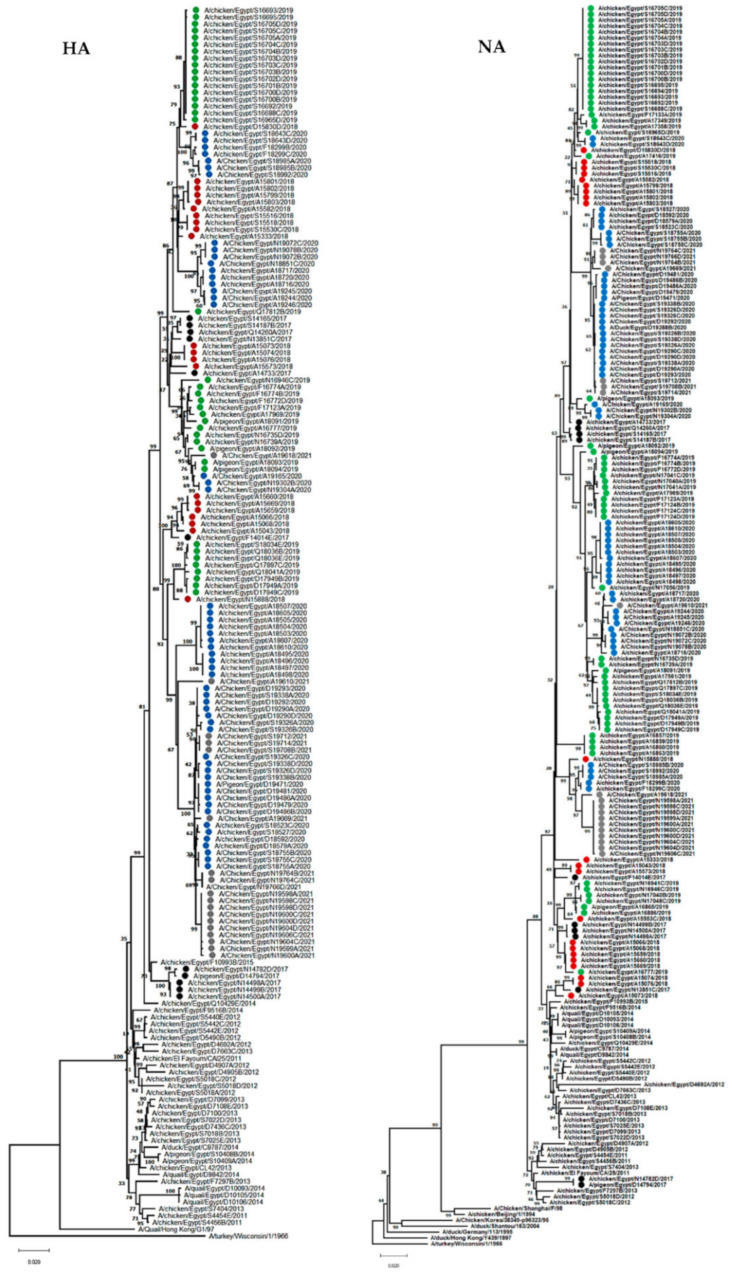
Neighbor joining phylogenetic trees of HA and NA of the detected H9N2 viruses in Egypt. The phylogenetic analysis was performed using MEGA version 7, and the evolutionary distances were computed using the Kimura 2-parameter method. H9N2 viruses sequenced specifically for this study are labeled with colored circles (2017 black, 2018 red, 2019 green, 2020 blue, and 2021 gray).

The HA genes of Egyptian H9N2 isolates from 2017–2021 showed a higher identity to H9N2 viruses isolated previously from Egypt (Figure 2). Genetic analysis of HA sequences showed that the Egyptian H9N2 viruses had six N-linked glycosylation sites (N-XT/S motif, where X can be any amino acid except proline) at positions 29, 105, 141, 298, 305, and 492 (H9 numbering). However, viruses A/chicken/Egypt/S19326A/2020 (H9N2) and A/chicken/Egypt/S19326B/2020 (H9N2) had only five glycosylation sites. Glycosylation sites at positions 206 and 218 were absent in all isolates compared with G1-like viruses. Moreover, the characteristic glycosylation site detected at position 196, which was previously observed in three quail H9N2 viruses from Egypt [21], was not detected in any of the viruses analyzed. Several receptor binding sites (RBS) of the HA of H9N2 viruses at positions 158/166, 183/191, 189/197, 190/198, 224/232, 226/234, 227/235, and 228/236 (H3/H9 numbering), which are determinant factors associated with the ability of the virus to bind to specific host cellular receptors, were analyzed (Table S4). Briefly, all H9N2 isolates had N166 except for A/chicken/Egypt/A15659/2018, A/chicken/Egypt/A15660/2018, and A/chicken/Egypt/A15669/2018, which had S158/166 (H3/H9 numbering) previously observed in A/quail/Hong Kong/G1/97 (H9N2) (Table S4). In contrast, all analyzed viruses had H, T, N, L, I, and G at positions 183/191, 189/197, 224/232, 226/234, 227/235, and 228/236 (H3/H9 numbering), respectively. Moreover, V was detected at position 190/198 (H3/H9 numbering) in 12 chicken isolates, and 1 isolate from pigeon carried T versus 15 isolates from chickens. T190/198 (H3/H9 numbering) was previously observed in A/duck/Hong Kong/Y280/97(H9N2), and other analyzed viruses had A at this site. All analyzed H9N2 viruses had H183/191 and L226/234 (H3/H9 numbering), which confirms the ability of the virus to bind to the cellular receptor of human respiratory epithelial cells [60]. Genetic analysis of different antigenic sites in the HA gene of H9N2 viruses revealed the presence of many amino acid mutations at the antigenic epitope sites of the HA. Genetic analysis of the HA cleavage motif showed that A/chicken/Egypt/A16777/2019, A/chicken/Egypt/S18985A/2020, A/chicken/Egypt/S18985B/2020, and A/chicken/Egypt/S18992/2020 had KSSR*GLF, and only A/chicken/Egypt/D19290B/2020 had VSDR*GLF. The remaining strains of H9N2 viruses had RSSR*GLF, which is evidence of the low pathogenic nature of different H9N2 viruses isolated from the Middle East and Asia, and adaptation of these viruses to the chicken host [9,61,62].

#### 3.1.5. NP

The NP tree topology revealed that NP genes of the H9N2 isolates from 2017–2021 showed a high identity to those of H9N2 isolates previously isolated in Egypt, except A/chicken/Egypt/A14733/2017, which was closely related to H9N2 viruses isolated previously from pigeons in Egypt in 2014 [21]; A/pigeon/Egypt/S10408B/2014 (H9N2), and A/pigeon/Egypt/S10409A/2014 (H9N2) (Figure 1).

The NP gene of Egyptian H9N2 isolates had amino acid substitutions associated with mammalian host specificity, such as V33I, which was observed in three isolates, A/pigeon/Egypt/A16865/2019, A/chicken/Egypt/A16886/2019, and A/chicken/Egypt/S16693/2019, whereas I61L was detected in seven isolates. Furthermore, K was observed at position 214 in all viruses analyzed except for A/chicken/Egypt/A14733/2017, which had R (avian-like marker). The substitution of D for E (mammalian-like marker) at position 375 was observed in two of the analyzed viruses, (A/chicken/Egypt/S18643C/2020) and (A/chicken/Egypt/S18643D/2020). On the other hand, Q, which is a mammalian host-specific marker, was observed at position 398 in all isolates.

#### 3.1.6. NA

The phylogenetic tree showed that NA genes of the 173 H9N2 isolates were closely related to the H9N2 viruses isolated previously from Egypt (Figure 2). Genetic analysis of NA sequences showed that stalk deletion was not detected in any of the analyzed viruses. The substitution of R for K at position 292 (N2 numbering), associated with resistance to oseltamivir and zanamivir [63], was not observed in any of the isolates. Moreover, the substitution of H by Y at position 274, known to cause resistance to oseltamivir [64], was not detected in any of the analyzed viruses. An analysis of binding-pocket residues in the NA genes involved in interactions with antiviral drugs showed an absence of mutations. All Egyptian viruses had 119E, 198D, 222I, 274H, and 292R residues. Egyptian H9N2 viruses had NA genes with eight N-linked glycosylation sites. Briefly, at position 146 and 200, all analyzed sequences had glycosylation sites. Moreover, glycosylation sites at positions 44, 69, 61, and 86 were seen in all isolates except three, one, three, and two chicken isolates, respectively. Furthermore, the glycosylation site at position 234 was missing in 12 chicken isolates. On the other hand, the characteristic glycosylation site of H9N2 viruses at position 402 [65] was observed in all viruses analyzed except one pigeon isolate and 31 chicken H9N2 isolates. Genetic analysis of the hemadsorption sites (366–373, 399–403, and 431–433), which are located on the surface of the NA molecule, away from the neuraminidase enzyme active site, revealed the presence of the IKKDSRAG form in all isolates in sites 366–373, except A/chicken/Egypt/A16777/2019, which had IEKDSRAG, two chicken isolates having VKKDSRAG, and 11 chicken isolates having IKTDSRAG at sites 399–404. The DSDNWS form was observed in 87 isolates, and DIDNRS, DSDDWS, DSDNRS, DSDSWS, DSGNWS, DSGSWS, DSNNRS, and DSNNWS forms were found in 10, 4, 5, 25, 1, 3, 3, and 35 isolates, respectively. At sites 431–433, three forms, PHE, PQE, and PRE, were observed in 34, 127, and 12 isolates, respectively.

#### 3.1.7. M

Phylogenetic analysis revealed that M genes of Egyptian isolates showed higher similarity with previously isolated H9N2 viruses from Egypt (Figure 1). Analysis of the M2 protein of Egyptian H9N2 isolates revealed the presence of amino acid substitutions associated with virulence, such as S and P at positions 64 and 69, which were detected in all viruses. On the other hand, the substitution of V for I at position 15 of the M1 protein, which is a mammalian host-specific marker, was observed in all viruses analyzed. Other host-specific markers of M1 were avian-like. Several amino acid substitutions associated with mammalian host specificity were observed in the M2 protein, such as the substitution of E for G at position 16, detected in all isolates except one isolate (A/chicken/Egypt/A17561/2019) that had V at this position. The substitution of S for N at position 20 was observed in only 11 isolates. Furthermore, V was found at position 28 in all viruses analyzed, except 15 isolates that had I. However, F55 was detected in all viruses analyzed except for three, A/chicken/Egypt/S18985A/2020, A/chicken/Egypt/S18985B/2020, and A/chicken/Egypt/S18992/2020, which had L (avian-like marker) at this position. All viruses analyzed revealed that the M2 protein had L, A, S, and G at positions 26, 30, 31, and 34, respectively, which is associated with the absence of resistance to amantadine. In addition to V27 that was observed in 12 viruses, I was observed in all isolates. However, 11 viruses had N at position 27, whereas S and T were observed in ten and three of the analyzed viruses, respectively.

#### 3.1.8. NS

The NS genes of Egyptian H9N2 isolates from 2017 to 2021 were highly related to two H9N2 viruses previously isolated from pigeons in Egypt in 2014 [21], A/pigeon/Egypt/S10408B/2014 (H9N2), and A/pigeon/Egypt/S10409A/2014 (H9N2), which were closely related to the NS of an A/tufted duck/Republic of Georgia/1/2012(H2N3) isolate and not to the NS of circulating Egyptian H9N2 viruses from 2010–2014 (Figure 1).

Genetic analysis of the NS1 protein of Egyptian isolates revealed that all viruses analyzed had S at position 42, which is a virulence marker [53] (Table 1). The substitution of D for E at position 92, which is a virulence determinant, was observed in three isolates, (A/chicken/Egypt/Q18041A/2019), (A/chicken/Egypt/D18579A/2020), and (A/chicken/Egypt/D18592/2020). However, 16 isolates had G at this position. The C-terminal PDZ-binding motif (X-S/T-X-V) of the NS1 protein was observed in 23 isolates in the form of ESEV, but the KSEV motif was not detected. The NS2 protein of all isolates had no residues associated with virulence at positions 31 and 56. The substitution of E for K/R at position 227 in NS1, which is associated with mammalian host specificity [66], has not been observed in any of the isolates (Table S3).

### 3.2. Genesis of Egyptian H9N2 Viruses

**Figure 3:**
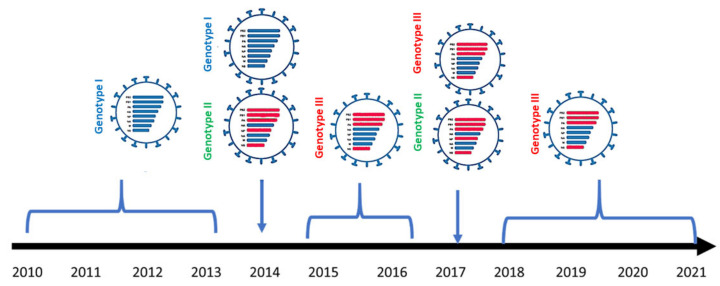
Schematic diagram showing genotypes and reassortment events of Egyptian H9N2 viruses by year (from 2010 to 2021). Blue bars indicate the first introduction form of H9N2 into Egypt, and the red bars indicate the segments that are derived from other subtypes of the Eurasian low pathogenic AI virus pool.

As revealed by our continuous, active surveillance of AI viruses in Egypt from 2010–2013, only one form of H9N2 was detected (designated as genotype I). In 2014, two reassortant H9N2 viruses (designated as genotype II) were detected in pigeons with five genes (PB2, PB1, PA, NP, and NS) from Eurasian AIVs circulating in wild birds and HA, NA, and M genes from the endemic Egyptian H9N2 viruses (genotype I). Other H9N2 isolates in the same year (2014) were similar to the previously detected viruses in 2010 (genotype I). In the period from 2015–2016, another reassortant form (designated as genotype III) was detected with four genes (PB2, PB1, PA, NS) from two previously characterized reassortant H9N2 viruses (genotype II) and HA, NP, NA, and M genes from the genotype I. The ancestor constellation form of 2010 (genotype I) was not identified after 2014. In 2017, both new reassortant forms (genotypes II and III) were identified. From 2018–2021, only one reassortant form (genotype III) of H9N2 was characterized (Figure 3). Of the 173 H9N2 isolates of the current study, only A/chicken/Egypt/A14733/2017 (H9N2) is related to genotype II, and the remaining 172 H9N2 viruses are related to genotype III.

### 3.3. Virological Characteristics and Pathogenicity of the Most Dominant Genotypes of Egyptian H9N2 Viruses

Based on genetic analysis, genotypes I and III were the most commonly detected viruses in Egypt. We compared the virological features and pathogenicity of two representative H9N2 viruses from genotypes I and III detected in Egypt to determine the effect of a reassortment event on virus behavior.

#### 3.3.1. Growth Kinetics of Egyptian H9N2 Viruses in Mammalian Cells and SPF-ECEs

**Figure 4:**
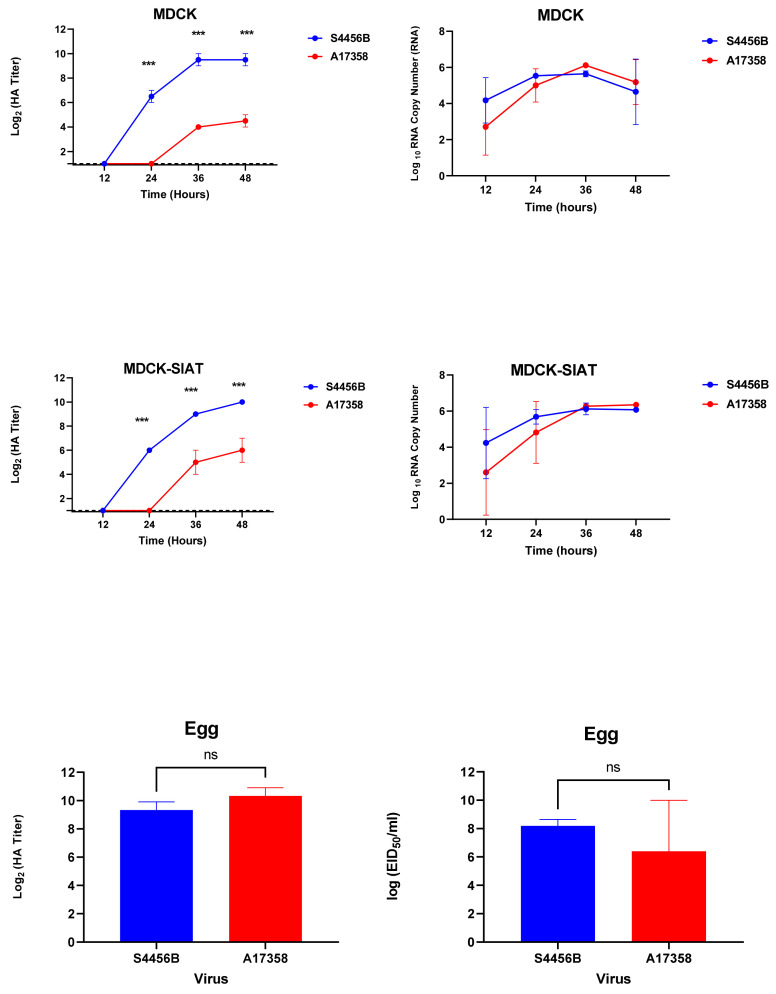
Growth kinetics of the two forms of Egyptian H9N2 viruses in MDCK, MDCK-SIAT, and SPF-ECEs. The cells were infected with the virus at an MOI of 0.01. At the time points indicated, the supernatant was taken and titrated by HA assay and TaqMan Real-Time PCR based on the measurement of M gene copies at different time points. The collected allantoic fluids of infected eggs with two viruses were titrated by the HA assay and EID50/mL. The significant differences are indicated (*** = *p* < 0.001 and non-significant = ns).

To evaluate the growth kinetics of the two most dominant forms of the Egyptian H9N2 AI viruses (genotypes I (S4456B virus) and III (A17358 virus)), MDCK and MDCK-SIAT cells were inoculated at an equal MOI of 0.01. The supernatants of infected cells were collected at specific time points and titrated by HA assays and TaqMan™ Real-Time PCR. The S4456B virus had the most significant HA titers at 24, 36, and 48 hpi in the supernatant harvested from infected MDCK and MDCK-SIAT cells, compared to the A17358 virus (*p* < 0.001). Furthermore, the growth of the two H9N2 viruses was compared in SPF-ECEs. Although the two viruses showed efficient replication in SPF-ECEs, no significant differences were observed between the two tested viruses in eggs (*p* > 0.05) (Figure 4).

#### 3.3.2. Viral Replication and Pathogenicity in Chickens and Ducks

**Figure 5:**
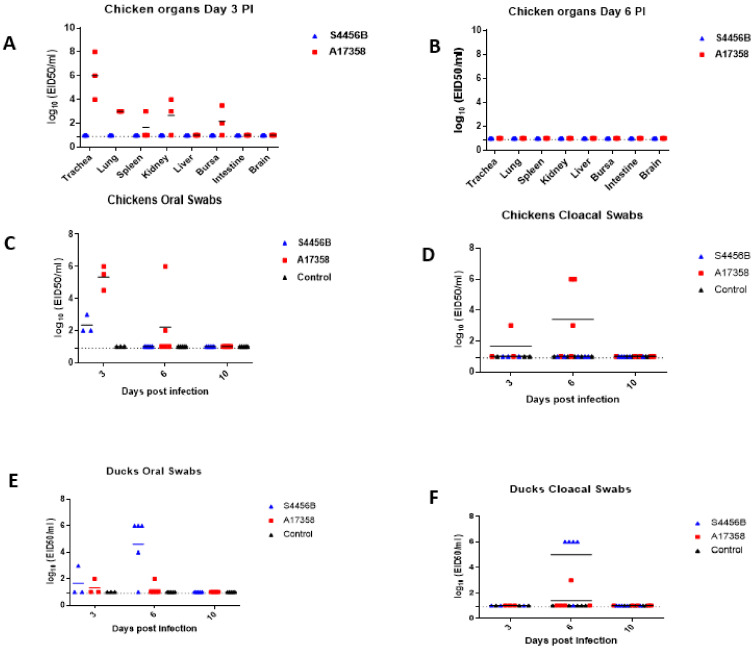
Replication of the two forms of the Egyptian H9N2 virus in infected chickens and ducks. Replication of both viruses in different organs of infected chicken at days 3 PI (**A**) and 6 PI (**B**). Oral (**C**) and cloacal (**D**) swabs at days 3, 6, and 10 PI were titrated by EID50. Oral and cloacal swabs were collected from ducks infected with the two viruses and from a control group at days 3, 6, and 10 PI. Oral and cloacal swabs were titrated by EID50 (**E**,**F**), respectively. The limit of virus detection is indicated by the dotted line.

The A17358 virus was detected on day 3 PI in multiple organs of infected chickens, but not in the intestine and brain, whereas the S4456B virus was not detected in any of the organs of the infected chickens (Figure 5A, Table S6). The highest viral titer of the A17358 virus in different organs was in the trachea (6 EID50/mL), followed by the lungs (3 EID50/mL) (Figure 5A). The viral titer in the organs collected from inoculated chickens with both viruses was not detected on day 6 PI (Figure 2, Table S6). Oral swabs collected from chickens inoculated with the A17358 and S4456B viruses on day 3 PI showed viral titers ranging from 4.5 to 6 EID50 and 2 to 3 EID50, respectively (Figure 5C, Table S7). On day 6, the A17358 virus was detected in two of three oral swabs, whereas the S4456B virus was not detected. Chickens infected with the A17358 virus shed the virus in cloacal swabs on day 3 and 6 PI, whereas other chickens infected with the S4456B virus did not shed the virus in the collected cloacal swabs. The two viruses were not detected in oral or cloacal swabs at day 10 PI (Figure 5C,D). To determine the seroconversion of chickens infected with H9N2 viruses, sera samples collected two weeks post-infection were titrated by HI assay against both viruses. Sera collected from chickens inoculated with the A17358 virus showed 12 log2 HI titer against a homologous virus, and sera collected from chickens infected with the S4456B virus had 8 log2 HI titer.

None of the ducks infected with both forms of the H9N2 virus showed any mortality or clinical signs. The S4456B virus was not detected in collected organs, and the A17358 virus was detected in only one duck trachea at day 3 PI (Table S6). Furthermore, the two viruses were not detected in any organ at day 6 PI (Table S6). Only one duck from each infected group with each form of the H9N2 virus shed the virus in oral swabs on day 3 PI (Figure 5, Table S7). On day 6, the S4456B virus was detected in both oral and cloacal swabs in most infected ducks, whereas only one of the ducks infected with the A17358 virus shed the virus in oral and cloacal swabs. The two Egyptian H9N2 viruses were not detected either in oral or cloacal swabs on day 10 PI. Sera collected from the ducks of both groups showed limited HI titer of 3 log2 against both H9N2 viruses.

#### 3.3.3. Viral Replication and Pathogenicity in Mice

Following the nasal infection of mice with 10^7^ EID_50_ per mouse of the two forms of the Egyptian H9N2 virus, neither clinical signs nor any significant changes in body weight were observed for 14 days post-inoculation. The S4456B virus was detected in the lungs and livers of two infected mice and the brain of only one infected mouse on day 3 PI (Table 2). The A17358 virus was detected in the lungs of two infected mice and in the liver of only one infected mouse on day 3 PI (Table 2). On day 6 PI, of the three euthanized mice belonging to the group infected with the S4456B virus, only one mouse had 1.6 Log _10_ RNA copy number in the lungs, and the virus was not detected in other organs. In addition, no viral RNA was detected in the organs of mice infected with the A17358 virus on day 6 PI (Table 2). An analysis of collected sera after 2 weeks PI by HI revealed the seroconversion of mice infected with the S4456B virus with 6.6 log_2_ HI titer against a homologous virus. There was no evidence of seroconversion of infected mice with the A17358 virus.

### 3.4. Immunogenicity of AI H9N2 Inactivated Vaccine in SPF Chickens

**Figure 6:**
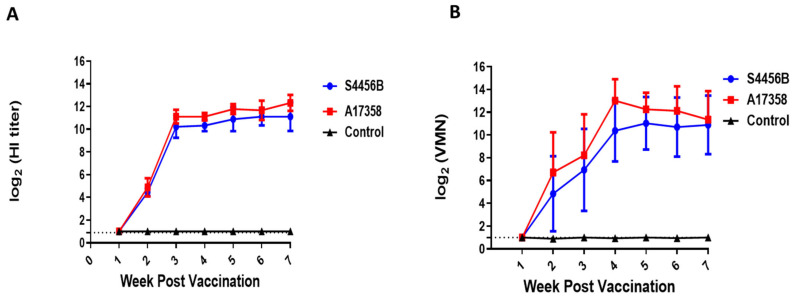
Weekly log2 antibody titers of SPF chicken vaccinated with A17358/H9N2 inactivated vaccine and unvaccinated control chickens against AI viruses A17358 and S4456B. (**A**) HI assay, (**B**) VMN assay. The detection limit of each assay is indicated by a dotted line.

Sera collected from ten 2-week-old SPF chickens showed no reaction with the S4456B /H9N2 and A17358/H9N2 viruses. All chickens vaccinated with the inactivated A17358 vaccine developed a significant antibody titer at 3 weeks post-vaccination (wpv) by both HI and VMN assays, and the titer increased over time through 4 wpv. No significant difference was detected in the reactivity of the antibodies of the A17358 vaccine against homologous (A17358) and heterologous (S4456B) viruses by either the HI or VMN assays. Sera collected from unvaccinated chickens had ≤2 log_2_ titers against the two H9N2 antigens over the seven weeks of the evaluation period of the immunogenicity of the vaccine, which is described as a nonspecific titer (Figure 6).

## 4. Discussion

H9N2 viruses are important pathogens for several reasons. Although they are less pathogenic in domestic poultry, H9N2 infections lead to economic losses, especially in breeder and layer flocks, and weaken birds’ immune systems, opening the door to infections with other pathogens with more severe consequences [67]. H9N2 are zoonotic viruses, with at least 72 confirmed human cases [68]. Importantly, H9N2 lend their internal gene constellation to more problematic AI subtypes, such as HPAI H5, H10Nx, and H7N9 [69,70,71].

After its first introduction into Egyptian domestic poultry in 2010, the AI H9N2 virus became endemic in Egyptian domestic poultry in different geographical regions across the country. This happened at the time when clade 2.2.1 H5N1 viruses were also enzootic, hence co-circulation and co-infection were reported [20]. In 2014, novel reassortant H9N2 viruses were detected in pigeons in Egypt that had three genes (HA, NA, and M) from the endemic H9N2 viruses in Egypt and five genes (PB2, PB1, PA, NP, and NS) from Eurasian AIVs circulating in wild birds [21]. As of 2017 (the year when H5N8 was first detected in domestic poultry in Egypt), the co–circulation of H9N2 and H5N8 in many poultry populations increased the possibility of a novel reassortment between the two subtypes that may lead to the emergence of new viruses with pandemic potential. Recently in Egypt, novel H5N2 reassortants between the Egyptian HPAI H5N8 and LPAI H9N2 viruses have been detected [72,73].

In Section 1 of the current study, we determined the genetic evolution of AI H9N2 viruses detected in domestic poultry in Egypt (*n* = 173 full genome sequences) from 2017 to 2021. Phylogenetic analysis of the HA indicated all sequences of Egyptian H9N2 viruses from 2017–2021 were grouped into G1-like H9N2 viruses previously detected in Egypt. A previous study reported that Egyptian H9N2 viruses before 2016 showed a close genetic relationship in the HA segment to those viruses from the Middle East grouped in lineage A of G1-like H9N2 viruses [72].

The HAs of Egyptian H9N2 viruses had K/RSSR*GLF cleavage motifs at the HA1-HA-2 connecting peptide, which is evidence of the low pathogenic nature of different H9N2 viruses isolated from the Middle East and Asia and the adaptation of these viruses to the chicken host [9,61,62].

Phylogenetic analysis of internal segments (PB2, PB1, PA, NP, NS) of Egyptian H9N2 viruses showed that they are heterogenous, and, accordingly, we classified Egyptian H9N2 viruses into three different genotypes (I, II, and III). Genotype I was detected in Egypt from 2010–2013. In 2014, genotype II was detected in only two reassortant H9N2 viruses in pigeons that had five segments (PB2, PB1, PA, NP, NS) from Eurasian AIVs circulating in wild birds with HA, NA, and M genes from genotype I. In the period from 2015–2016, another reassortant form (genotype III) was detected that had four genes (PB2, PB1, PA, NS) from genotype II and HA, NP, NA, and M genes from genotype I. Genotype replacement was observed in the current study. The ancestor constellation form of 2010 (genotype I) was not identified after 2014. In 2017, both new reassortant forms (genotypes II and III) were identified. From 2018–2021, only one reassortant form (genotype III) of H9N2 was identified. H9N2 viruses have been detected in mallards during AI surveillance study in live wild bird markets in Egypt from 2014–2016 [23]. During this study, several LPAIVs were detected, including H7N3, H7N9, H3N6, and H10N6, suggesting that reassortment events likely occurred there.

In the second part of this study, we compared the virological features of the two most dominant genotypes of Egyptian H9N2 viruses in vitro and in vivo to determine the effect of reassortment on virus characteristics. Our results indicated that both genotypes replicated well in mammalian cells, and genotype I had a higher replication rate than genotype III in mammalian cells. Generally, the virulence of AIVs is correlated with the mortality rate, morbidity signs, and replication titer in different organs in animals. Concerning pathogenicity in chickens and ducks, both genotypes of the Egyptian H9N2 virus generally did not show any clinical signs post-infection, although the behavior of both viruses in the two infected hosts is completely different. Genotype III can replicate well in chickens, but not genotype I, which is attributed to reassortment or acquiring an adaptive mutation. Contrary to previous studies in Egypt reporting that infection of the Egyptian H9N2 virus in broilers showed pathogenic symptoms in infected chickens [74,75], our results indicated low pathogenicity of AI H9N2 from Egypt. In the present investigation, it was noticed that chickens inoculated with both genotypes seroconverted with high HI within two weeks post-infection, and limited titer was detected in ducks.

The two genotypes of the Egyptian H9N2 virus did not show any significant changes in the body weight of infected mice over 14 days post-inoculation and showed limited replication titers in the lungs of some infected mice. A previous study indicated that H9N2 viruses need to be adapted in mice to acquire severity and cause systemic infection [76]. Analyzing genetic markers associated with the virulence of both tested H9N2 viruses indicated that the two viruses had I504V in PB2 [31], I127V in PA [49], P64S and L69P in M2 [49], and A42S in NS1 [53]. By analyzing the genetic markers associated with host range specificity, the A17358 virus had specific M64T in PB2 [36] and E382D in PA [35,57], previously known as mammalian preference markers.

Some of the genotypic markers in Egyptian H9N2 viruses still need to be investigated in further studies to determine the role of each. This could further shed light on the variation in pathogenicity in mammalian and avian hosts, as we observed. Overall, this stresses the need for biological characterization of the different detected genotypes of H9N2 viruses.

Some genetic markers that are known to increase transmission of AIVs to mammalian hosts were detected in Egyptian H9N2 viruses. Between 2011 and 2015, H9N2 viruses had L234 and H191, which are associated with the alteration of the HA affinity from avian α-2,3 SAs to human α-2,6 SA receptors. Genetic evolution played a role in increasing human infections of H9N2, especially the change of amino acid from glutamine (Q) to leucine (L) at position 226 in the HA RBS that is responsible for binding to human type α2,6 sialic acid receptor and increased H9N2 transmission in ferrets. All Egyptian H9N2 isolates had H191 and L234 that were previously detected in human H3N2 isolates [60].

Egypt has adopted vaccination strategy as a key approach to control endemic AIVs. Vaccination against H9N2 viruses has been implemented in Egypt since 2012. Different types of commercial inactivated H9N2 vaccines are used in Egypt that are based on either local strains of genotype I (A/chicken/Egypt/114940v/2011) or other H9N2 strains from the Middle East (A/chicken/Saudi Arabia/CP7/1998, and A/chicken/Iran/av1221/1998). Although H9N2 viruses detected in Egypt have shown wide antigenic variability, commercial vaccines used in Egypt are generally not updated based on antigenically drifted viruses. In the current study, at least 14 H9N2 viruses were isolated from flocks that were vaccinated with H9N2 vaccines (Table S1). The evaluation of commercially used vaccines in Egypt is recommended, and vaccines should be updated. The results of our study indicated that genotype I and genotype III have good cross-reactivity.

Reassortment events leading to novel influenza virus species are always of major concern. This is particularly true for H9N2 viruses, which are among the most important AIVs. Hence, continuous monitoring of these viruses through systematic surveillance programs is a must. Furthermore, understanding the genetic and antigenic characteristics, pathogenicity, host range, and transmissibility of circulating H9N2 viruses will aid in risk assessment exercises that are required for influenza pandemic preparedness.

## Figures and Tables

**Table 1:** Analysis of genetic markers associated with virulence in the viral PB2, PB1-F2, PB1, PA, NP, M1, M2, NS1, and N S2 proteins of the 173 Egyptian H9N2 viruses isolated from poultry in Egypt from 2017 to 2021.

Protein	Site	Avirulent	Virulent	Subtypes Tested	Total Egyptian H9N2 (2017–2021) = 173	References
PB2	627	E	K	H9N2	E (172), K (1)	[37,39]
	147	M	L	H9N2	I (155), N/A (18)	[37]
	250	V	G	H9N2	V (155), N/A (18)	[37]
	292	I	V	H7N9	I (101), V (57), N/A (15)	[40]
	504	I	V	H1N1	V (170), N/A (3)	[31]
	588	A	V	H5N1 H7N9	A (169), V (4)	[41,42]
	701	D	N	H1N1, H5N1	D (173)	[38,43]
	404	F	L	H9N2	F (163), N/A (10)	[44]
	591	Q	K	H7N9	Q (171), L (2)	[45]
PB1	317	M/V	I	(H5N1, H9N2, H7N2, H7N7), H5N1	M (165), I (5), N/A (3)	[34,46]
	622	D	G	H5N1	G (171), S (1), N/A (1)	[47]
PB1-F2	66	N	S	H5N1	N (120), K (33), N/A (13) 166	[48]
PA	127	I	V	H5N1	V (155), N/A (18)	[49]
	383	N	D	H5N1	D(173)	[50]
	224	S	P	H5N1	S (156), N/A (17)	[50]
	550	I	L	H1N1	L (173)	[31]
HA	Cleavage site	Monobasic	Multibasic	H5N1	KSSR*GLF (4), VSDR*GLF (1), RSSR*GLF (168)	[51]
NP	286	A	V	H7N9	A (173)	[52]
	437	T	M	H7N9	T (173)	
M2	64	P	S/A/F	H5N1	S (173)	[49]
	69	L	P	H5N1	P (173)	[49]
NS1	42	A/P	S	H5N1	S (172), N/A (1)	[53]
	92	D	E	H5N1	D (170), E (3)	[46]
	103	F	L	H3N2	F (173)	[54]
	106	M	I	H3N2	M (173)	[54]
	149	V	A	H5N1	A (173)	[55]
	189	D/G	N	H5N1	D (173)	[56]
NS2	31	M	I	H5N1	M (173)	[56]
	56	H/L	Y	H5N1	H (173)	[56]

N/A: not applicable due to partial sequencing.

**Table 2:** Viral loads (Log 10 RNA copy number) of H9N2 viruses in different organs of infected mice with a dose of 10^7^ EID50/mouse.

Organs	DPI	S4456B	A17358
Lung	3	3.6 ± 1 (2/3) *	1.96 ± 0.65 (2/3)
Brain		8.7(1/3)	ND
Kidney		ND	ND
Liver		2.55 ± 1 (2/3)	1(1/3)
Lung	6	1.6 (1/3)	ND
Brain		ND	ND
Kidney		ND	ND
Liver		ND	ND

* Log 10 RNA copy number ± SD (number of positives/total number). ND: Not detected.

## Data Availability

The data presented in this study are available in the manuscript and supplementary material. All sequences presented in this study are openly available in GenBank under the accession numbers listed in Table S2.

## Supplementary Materials

The following supporting information can be downloaded at: https://www.mdpi.com/article/10.3390/v14071484/s1, Table S1: Summarized profile of Egyptian AIV H9N2 isolates used in this study; Table S2: Summary of the Egyptian AIV H9N2 genes analyzed in the current study with their accession numbers in GenBank; Table S3: Analysis of genetic markers associated with host range specificity in the PB2, PB1-F2, PB1, PA, NP, M1, M2, and NS1 proteins in 173 H9N2 viruses isolated from poultry in Egypt (the avian or mammalian-preference markers are shown); Table S4: Comparison of the Glycosylation sites, RBS, and antigenic sites of the HA of H9N2 viruses isolated from poultry in Egypt between 2017 and 2021; Table S5: Comparison of the Glycosylation Sites of the NA of H9N2 viruses isolated from poultry in Egypt between 2017–2021; Table S6: Titers of A/chicken/Egypt/S4456B/2011(H9N2) and A/chicken/Egypt/A17358/2019 (H9N2) viruses in organs obtained from infected chicken and duck groups; Table S7: Titers of A/chicken/Egypt/S4456B/2011(H9N2) and A/chicken/Egypt/A17358/2019 (H9N2) viruses in swabs obtained from infected chicken and duck groups.
