# Supplementary material for: Insights into Genetic Characteristics and Virological Features of Endemic Avian Influenza A (H9N2) Viruses in Egypt from 2017–2021
Source: Viruses. 2022 Jul 6;14(7):1484. doi: 10.3390/v14071484 (PMC9321558; doi:10.3390/v14071484)
Supplement: Supplementary file 1 [file viruses-14-01484-s001.zip › viruses-1728153-Supplementary Materials.pdf]

Table S1. Summarized profile of Egyptian AIV H9N2 isolates used in this study.

S.N	H9N2 Isolate	Genotype	Governorate	Host	Health Status	Sequence	Collection Site	Collection Date	Vaccination Status
1	A/chicken/Egypt/N13851C/2017	III	Menia	Chicken	Healthy	Full genome	House	15/03/2017	Unknown
2	A/chicken/Egypt/F14014E/2017	III	Fayoum	Chicken	Dead	Full genome	Farm	25/04/2017	H9N2 VACCINATED
3	A/chicken/Egypt/S14165/2017	III	Sharqeia	Chicken	Healthy	Full genome	Market	05/06/2017	Unknown
4	A/chicken/Egypt/S14187B/2017	III	Sharqeia	Chicken	Healthy	Full genome	Farm	05/06/2017	Unknown
5	A/chicken/Egypt/Q14260A/2017	III	Kalyobiya	Chicken	Dead	Full genome	Farm	08/07/2017	H5N1, H9N2 VACCINATED
6	A/chicken/Egypt/N14498A/2017	III	Menia	Chicken	Healthy	Full genome	House	11/09/2017	Unknown
7	A/chicken/Egypt/N14499B/2017	III	Menia	Chicken	Healthy	Full genome	House	11/09/2017	Unknown
8	A/chicken/Egypt/N14500A/2017	III	Menia	Chicken	Healthy	Full genome	House	11/09/2017	Unknown
9	A/chicken/Egypt/A14733/2017	II	Assiut	Chicken	Healthy	Full genome	Market	08/11/2017	Unknown
10	A/chicken/Egypt/N14782D/2017	III	Menia	Chicken	Healthy	Full genome	House	13/11/2017	Unknown
11	A/pigeon/Egypt/D14794/2017	III	Dakahliya	Pigeon	Healthy	Full genome	Market	18/11/2017	Unknown
12	A/chicken/EGYPT/A15043/2018	III	Assiut	Chicken	Healthy	Full genome	Market	15/01/2018	Unknown
13	A/chicken/EGYPT/A15066/2018	III	Assiut	Chicken	Healthy	Full genome	Market	15/01/2018	Unknown
14	A/chicken/EGYPT/A15068/2018	III	Assiut	Chicken	Healthy	Full genome	Market	15/01/2018	Unknown
15	A/chicken/EGYPT/A15073/2018	III	Assiut	Chicken	Healthy	Full genome	Market	15/01/2018	Unknown
16	A/chicken/EGYPT/A15074/2018	III	Assiut	Chicken	Healthy	Full genome	Market	15/01/2018	Unknown
17	A/chicken/EGYPT/A15076/2018	III	Assiut	Chicken	Healthy	Full genome	Market	15/01/2018	Unknown
18	A/chicken/EGYPT/A15333/2018	III	Assiut	Chicken	Healthy	Full genome	Market	31/03/2018	Unknown
19	A/chicken/EGYPT/S15516/2018	III	Sharqeia	Chicken	Healthy	Full genome	Market	06/05/2018	Unknown
20	A/chicken/EGYPT/S15518/2018	III	Sharqeia	Chicken	Healthy	Full genome	Market	06/05/2018	Unknown
21	A/chicken/EGYPT/S15530C/2018	III	Sharqeia	Chicken	Healthy	Full genome	Farm	06/05/2018	Unknown
22	A/chicken/EGYPT/A15553C/2018	III	Assiut	Chicken	Healthy	Full genome	Farm	14/05/2018	H9N2, NDV vaccinated
23	A/chicken/EGYPT/A15573/2018	III	Assiut	Chicken	Healthy	Full genome	Market	14/05/2018	H9N2, NDV vaccinated
24	A/chicken/EGYPT/A15582/2018	III	Assiut	Chicken	Healthy	Full genome	Market	14/05/2018	H9N2, NDV vaccinated
25	A/chicken/EGYPT/A15659/2018	III	Assiut	Chicken	Healthy	Full genome	Market	18/06/2018	Unknown
26	A/chicken/EGYPT/A15660/2018	III	Assiut	Chicken	Healthy	Full genome	Market	18/06/2018	Unknown
27	A/chicken/EGYPT/A15669/2018	III	Assiut	Chicken	Healthy	Full genome	Market	18/06/2018	Unknown

28	A/chicken/EGYPT/A15799/2018	III	Assiut	Chicken	Healthy	Full genome	Market	10/07/2018	Unknown
29	A/chicken/EGYPT/A15801/2018	III	Assiut	Chicken	Healthy	Full genome	Market	10/07/2018	Unknown
30	A/chicken/EGYPT/A15802/2018	III	Assiut	Chicken	Healthy	Full genome	Market	10/07/2018	Unknown
31	A/chicken/EGYPT/A15803/2018	III	Assiut	Chicken	Healthy	Full genome	Market	10/07/2018	Unknown
32	A/chicken/EGYPT/D15830D/2018	III	Dakahliya	Chicken	Healthy	Full genome	House	23/07/2018	Unknown
33	A/chicken/EGYPT/N15888/2018	III	Menia	Chicken	Healthy	Full genome	Market	13/08/2018	Unknown
34	A/chicken/Egypt/S16688C/2019	III	Sharqeia	Chicken	Sick	Full genome	Farm	06/02/2019	Unknown
35	A/chicken/Egypt/S16692/2019	III	Sharqeia	Chicken	Healthy	Full genome	Market	06/02/2019	Unknown
36	A/chicken/Egypt/S16693/2019	III	Sharqeia	Chicken	Healthy	Full genome	Market	06/02/2019	Unknown
37	A/chicken/Egypt/S16694/2019	III	Sharqeia	Chicken	Healthy	Full genome	Market	06/02/2019	Unknown
38	A/chicken/Egypt/S16695/2019	III	Sharqeia	Chicken	Healthy	Full genome	Market	06/02/2019	Unknown
39	A/chicken/Egypt/S16700B/2019	III	Sharqeia	Chicken	Sick	Full genome	Farm	06/02/2019	Unknown
40	A/chicken/Egypt/S16700D/2019	III	Sharqeia	Chicken	Sick	Full genome	Farm	06/02/2019	Unknown
41	A/chicken/Egypt/S16701B/2019	III	Sharqeia	Chicken	Sick	Full genome	Farm	06/02/2019	Unknown
42	A/chicken/Egypt/S16702D/2019	III	Sharqeia	Chicken	Healthy	Full genome	Farm	06/02/2019	Unknown
43	A/chicken/Egypt/S16703B/2019	III	Sharqeia	Chicken	Healthy	Full genome	Farm	06/02/2019	Unknown
44	A/chicken/Egypt/S16703C/2019	III	Sharqeia	Chicken	Healthy	Full genome	Farm	06/02/2019	Unknown
45	A/chicken/Egypt/S16703D/2019	III	Sharqeia	Chicken	Healthy	Full genome	Farm	06/02/2019	Unknown
46	A/chicken/Egypt/S16704A/2019	III	Sharqeia	Chicken	Healthy	Full genome	Farm	06/02/2019	Unknown
47	A/chicken/Egypt/S16704B/2019	III	Sharqeia	Chicken	Healthy	Full genome	Farm	06/02/2019	Unknown
48	A/chicken/Egypt/S16704C/2019	III	Sharqeia	Chicken	Healthy	Full genome	Farm	06/02/2019	Unknown
49	A/chicken/Egypt/S16705A/2019	III	Sharqeia	Chicken	Healthy	Full genome	Farm	06/02/2019	Unknown
50	A/chicken/Egypt/S16705C/2019	III	Sharqeia	Chicken	Healthy	Full genome	Farm	06/02/2019	Unknown
51	A/chicken/Egypt/S16705D/2019	III	Sharqeia	Chicken	Healthy	Full genome	Farm	06/02/2019	Unknown
52	A/chicken/Egypt/N16735D/2019	III	Menia	Chicken	Healthy	Full genome	House	21/02/2019	Unknown
53	A/chicken/Egypt/N16739A/2019	III	Menia	Chicken	Healthy	Full genome	House	22/02/2019	Unknown
54	A/chicken/Egypt/F16772D/2019	III	Fayoum	Chicken	Dead	Full genome	Farm	25/02/2019	Unknown
55	A/chicken/Egypt/F16774A/2019	III	Fayoum	Chicken	Dead	Full genome	Farm	25/02/2019	Unknown
56	A/chicken/Egypt/F16774B/2019	III	Fayoum	Chicken	Dead	Full genome	Farm	25/02/2019	Unknown
57	A/chicken/Egypt/A16777/2019	III	Assiut	Chicken	Healthy	Full genome	Market	27/02/2019	Unknown
58	A/chicken/Egypt/A16857/2019	III	Assiut	Chicken	Healthy	Full genome	Market	19/03/2019	Unknown

59	A/chicken/Egypt/A16859/2019	III	Assiut	Chicken	Healthy	Full genome	Market	19/03/2019	Unknown
60	A/chicken/Egypt/A16860/2019	III	Assiut	Chicken	Healthy	Full genome	Market	19/03/2019	Unknown
61	A/chicken/Egypt/A16863/2019	III	Assiut	Chicken	Healthy	Full genome	Market	19/03/2019	Unknown
62	A/pigeon/Egypt/A16865/2019	III	Assiut	Pigeon	Healthy	Full genome	Market	19/03/2019	Unknown
63	A/chicken/Egypt/A16886/2019	III	Assiut	Chicken	Healthy	Full genome	Market	19/03/2019	Unknown
64	A/chicken/Egypt/N16941C/2019	III	Menia	Chicken	Healthy	Full genome	Farm	26/03/2019	Unknown
65	A/chicken/Egypt/N16946C/2019	III	Menia	Chicken	Healthy	Full genome	Farm	26/03/2019	Unknown
66	A/chicken/Egypt/S16965D/2019	III	Sharqeia	Chicken	Healthy	Full genome	Farm	06/04/2019	Unknown
67	A/chicken/Egypt/N17040A/2019	III	Menia	Chicken	Healthy	Full genome	Farm	30/04/2019	Unknown
68	A/chicken/Egypt/N17040B/2019	III	Menia	Chicken	Healthy	Full genome	Farm	30/04/2019	Unknown
69	A/chicken/Egypt/N17041A/2019	III	Menia	Chicken	Healthy	Full genome	Farm	30/04/2019	Unknown
70	A/chicken/Egypt/N17041C/2019	III	Menia	Chicken	Healthy	Full genome	Farm	30/04/2019	Unknown
71	A/chicken/Egypt/N17048C/2019	III	Menia	Chicken	Healthy	Full genome	House	30/04/2019	Unknown
72	A/chicken/Egypt/N17056/2019	III	Menia	Chicken	Healthy	Full genome	Market	30/04/2019	Unknown
73	A/chicken/Egypt/F17123A/2019	III	Fayoum	Chicken	Sick	Full genome	Farm	30/05/2019	H9N2 VACCINATED
74	A/chicken/Egypt/F17124B/2019	III	Fayoum	Chicken	Sick	Full genome	Farm	30/05/2019	H9N2 VACCINATED
75	A/chicken/Egypt/F17124C/2019	III	Fayoum	Chicken	Sick	Full genome	Farm	30/05/2019	H9N2 VACCINATED
76	A/chicken/Egypt/F17124D/2019	III	Fayoum	Chicken	Sick	Full genome	Farm	30/05/2019	H9N2 VACCINATED
77	A/chicken/Egypt/F17133A/2019	III	Fayoum	Chicken	Sick	Full genome	Farm	25/05/2019	H9N2 VACCINATED
78	A/chicken/Egypt/A17349/2019	III	Assiut	Chicken	Healthy	Full genome	Market	31/07/2019	Unknown
79	A/chicken/Egypt/A17358/2019	III	Assiut	Chicken	Healthy	Full genome	Market	31/07/2019	Unknown
80	A/chicken/Egypt/A17416/2019	III	Assiut	Chicken	Healthy	Full genome	Market	19/08/2019	Unknown
81	A/chicken/Egypt/A17561/2019	III	Assiut	Chicken	Healthy	Full genome	Market	22/09/2019	Unknown
82	A/chicken/Egypt/Q17812B/2019	III	Kalyobiya	Chicken	Dead	Full genome	Farm	19/10/2019	H9N2 VACCINATED
83	A/chicken/Egypt/Q17897C/2019	III	Kalyobiya	Chicken	Dead	Full genome	Farm	02/11/2019	H5N1, H9N2 VACCINATED
84	A/chicken/Egypt/D17949A/2019	III	Dakahliya	Chicken	Healthy	Full genome	Farm	23/11/2019	Unknown
85	A/chicken/Egypt/D17949B/2019	III	Dakahliya	Chicken	Healthy	Full genome	Farm	23/11/2019	Unknown
86	A/chicken/Egypt/D17949C/2019	III	Dakahliya	Chicken	Healthy	Full genome	Farm	23/11/2019	Unknown
87	A/chicken/Egypt/A17969/2019	III	Assiut	Chicken	Healthy	Full genome	Market	23/11/2019	Unknown
88	A/chicken/Egypt/S18034E/2019	III	Sharqeia	Chicken	Dead	Full genome	Farm	07/12/2019	H5N1 Vaccinated
89	A/chicken/Egypt/Q18036B/2019	III	Kalyobiya	Chicken	Dead	Full genome	Farm	07/12/2019	H5N1, H9N2 VACCINATED

90	A/chicken/Egypt/Q18036E/2019	III	Kalyobiya	Chicken	Dead	Full genome	Farm	07/12/2019	H5N1, H9N2 VACCINATED
91	A/chicken/Egypt/Q18041A/2019	III	Kalyobiya	Chicken	Dead	Full genome	Farm	07/12/2019	H5N1 Vaccinated
92	A/pigeon/Egypt/A18091/2019	III	Assiut	Pigeon	Healthy	Full genome	Market	19/12/2019	Unknown
93	A/pigeon/Egypt/A18092/2019	III	Assiut	Pigeon	Healthy	Full genome	Market	19/12/2019	Unknown
94	A/pigeon/Egypt/A18093/2019	III	Assiut	Pigeon	Healthy	Full genome	Market	19/12/2019	Unknown
95	A/pigeon/Egypt/A18094/2019	III	Assiut	Pigeon	Healthy	Full genome	Market	19/12/2019	Unknown
96	A/chicken/Egypt/F18299B/2020	III	Fayoum	Chicken	Dead	Full genome	Farm	22/01/2020	Unknown
97	A/chicken/Egypt/F18299C/2020	III	Fayoum	Chicken	Dead	Full genome	Farm	22/01/2020	Unknown
98	A/chicken/Egypt/A18495/2020	III	Assiut	Chicken	Healthy	Full genome	Market	31/03/2020	Unknown
99	A/chicken/Egypt/A18496/2020	III	Assiut	Chicken	Healthy	Full genome	Market	31/03/2020	Unknown
100	A/chicken/Egypt/A18497/2020	III	Assiut	Chicken	Healthy	Full genome	Market	31/03/2020	Unknown
101	A/chicken/Egypt/A18498/2020	III	Assiut	Chicken	Healthy	Full genome	Market	31/03/2020	Unknown
102	A/chicken/Egypt/A18503/2020	III	Assiut	Chicken	Healthy	Full genome	Market	31/03/2020	Unknown
103	A/chicken/Egypt/A18504/2020	III	Assiut	Chicken	Healthy	Full genome	Market	31/03/2020	Unknown
104	A/chicken/Egypt/A18505/2020	III	Assiut	Chicken	Healthy	Full genome	Market	31/03/2020	Unknown
105	A/chicken/Egypt/A18507/2020	III	Assiut	Chicken	Healthy	Full genome	Market	31/03/2020	Unknown
106	A/chicken/Egypt/S18523C/2020	III	Sharqeia	Chicken	Healthy	Full genome	Farm	07/04/2020	Unknown
107	A/chicken/Egypt/S18527/2020	III	Sharqeia	Chicken	Healthy	Full genome	Market	07/04/2020	Unknown
108	A/chicken/Egypt/D18579A/2020	III	Dakahliya	Chicken	Healthy	Full genome	Farm	19/04/2020	Unknown
109	A/chicken/Egypt/D18592/2020	III	Dakahliya	Chicken	Healthy	Full genome	Market	19/04/2020	Unknown
110	A/chicken/Egypt/A18605/2020	III	Assiut	Chicken	Healthy	Full genome	Market	20/04/2020	Unknown
111	A/chicken/Egypt/A18607/2020	III	Assiut	Chicken	Healthy	Full genome	Market	20/04/2020	Unknown
112	A/chicken/Egypt/A18610/2020	III	Assiut	Chicken	Healthy	Full genome	Market	20/04/2020	Unknown
113	A/chicken/Egypt/S18643C/2020	III	Sharqeia	Chicken	Sick	Full genome	Farm	06/05/2020	Unknown
114	A/chicken/Egypt/S18643D/2020	III	Sharqeia	Chicken	Sick	Full genome	Farm	06/05/2020	Unknown
115	A/chicken/Egypt/A18716/2020	III	Assiut	Chicken	Healthy	Full genome	Market	31/05/2020	Unknown
116	A/chicken/Egypt/A18717/2020	III	Assiut	Chicken	Healthy	Full genome	Market	31/05/2020	Unknown
117	A/chicken/Egypt/A18720/2020	III	Assiut	Chicken	Healthy	Full genome	Market	31/05/2020	Unknown
118	A/chicken/Egypt/S18755A/2020	III	Sharqeia	Chicken	Sick	Full genome	Farm	10/06/2020	Unknown
119	A/chicken/Egypt/S18755B/2020	III	Sharqeia	Chicken	Sick	Full genome	Farm	10/06/2020	Unknown
120	A/chicken/Egypt/S18755C/2020	III	Sharqeia	Chicken	Sick	Full genome	Farm	10/06/2020	Unknown

121	A/chicken/Egypt/S18755D/2020	III	Sharqeia	Chicken	Sick	Full genome	Farm	10/06/2020	Unknown
122	A/chicken/Egypt/N18851C/2020	III	Menia	Chicken	Healthy	Full genome	Farm	23/06/2020	Unknown
123	A/chicken/Egypt/S18985A/2020	III	Sharqeia	Chicken	Healthy	Full genome	House	12/08/2020	Unknown
124	A/chicken/Egypt/S18985B/2020	III	Sharqeia	Chicken	Healthy	Full genome	House	12/08/2020	Unknown
125	A/chicken/Egypt/S18992/2020	III	Sharqeia	Chicken	Healthy	Full genome	Market	12/08/2020	Unknown
126	A/chicken/Egypt/N19072B/2020	III	Menia	Chicken	Healthy	Full genome	Farm	29/08/2020	Unknown
127	A/chicken/Egypt/N19072C/2020	III	Menia	Chicken	Healthy	Full genome	Farm	29/08/2020	Unknown
128	A/chicken/Egypt/N19074A/2020	III	Menia	Chicken	Healthy	Full genome	Farm	29/08/2020	Unknown
129	A/chicken/Egypt/N19078B/2020	III	Menia	Chicken	Healthy	Full genome	House	29/08/2020	Unknown
130	A/chicken/Egypt/A19165/2020	III	Assiut	Chicken	Healthy	Full genome	Market	22/09/2020	Unknown
131	A/chicken/Egypt/A19244/2020	III	Assiut	Chicken	Healthy	Full genome	Market	11/10/2020	Unknown
132	A/chicken/Egypt/A19245/2020	III	Assiut	Chicken	Healthy	Full genome	Market	11/10/2020	Unknown
133	A/chicken/Egypt/A19246/2020	III	Assiut	Chicken	Healthy	Full genome	Market	11/10/2020	Unknown
134	A/duck/Egypt/D19288B/2020	III	Dakahliya	Duck	Healthy	Full genome	House	25/10/2020	Unknown
135	A/chicken/Egypt/D19290A/2020	III	Dakahliya	Chicken	Healthy	Full genome	Farm	25/10/2020	Unknown
136	A/chicken/Egypt/D19290B/2020	III	Dakahliya	Chicken	Healthy	Full genome	Farm	25/10/2020	Unknown
137	A/chicken/Egypt/D19290C/2020	III	Dakahliya	Chicken	Healthy	Full genome	Farm	25/10/2020	Unknown
138	A/chicken/Egypt/D19290D/2020	III	Dakahliya	Chicken	Healthy	Full genome	Farm	25/10/2020	Unknown
139	A/chicken/Egypt/D19292/2020	III	Dakahliya	Chicken	Healthy	Full genome	Market	25/10/2020	Unknown
140	A/chicken/Egypt/D19293/2020	III	Dakahliya	Chicken	Healthy	Full genome	Market	25/10/2020	Unknown
141	A/chicken/Egypt/N19302B/2020	III	Menia	Chicken	Healthy	Full genome	Farm	26/10/2020	Unknown
142	A/chicken/Egypt/N19304A/2020	III	Menia	Chicken	Healthy	Full genome	Farm	26/10/2020	Unknown
143	A/chicken/Egypt/S19326A/2020	III	Sharqeia	Chicken	Healthy	Full genome	House	07/11/2020	Unknown
144	A/chicken/Egypt/S19326B/2020	III	Sharqeia	Chicken	Healthy	Full genome	House	07/11/2020	Unknown
145	A/chicken/Egypt/S19326C/2020	III	Sharqeia	Chicken	Healthy	Full genome	House	07/11/2020	Unknown
146	A/chicken/Egypt/S19326D/2020	III	Sharqeia	Chicken	Healthy	Full genome	House	07/11/2020	Unknown
147	A/chicken/Egypt/S19338A/2020	III	Sharqeia	Chicken	Healthy	Full genome	Farm	07/11/2020	Unknown
148	A/chicken/Egypt/S19338B/2020	III	Sharqeia	Chicken	Healthy	Full genome	Farm	07/11/2020	Unknown
149	A/chicken/Egypt/S19338D/2020	III	Sharqeia	Chicken	Healthy	Full genome	Farm	07/11/2020	Unknown
150	A/pigeon/Egypt/D19471/2020	III	Dakahliya	Pigeon	Healthy	Full genome	Market	21/12/2020	Unknown
151	A/chicken/Egypt/D19479/2020	III	Dakahliya	Chicken	Healthy	Full genome	Market	21/12/2020	Unknown

152	A/chicken/Egypt/D19481/2020	III	Dakahliya	Chicken	Healthy	Full genome	Market	21/12/2020	Unknown
153	A/chicken/Egypt/D19486A/2020	III	Dakahliya	Chicken	Healthy	Full genome	Farm	21/12/2020	Unknown
154	A/chicken/Egypt/D19486B/2020	III	Dakahliya	Chicken	Healthy	Full genome	Farm	21/12/2020	Unknown
155	A/chicken/Egypt/N19598A/2021	III	Menia	Chicken	Healthy	Full genome	Farm	24/01/2021	Unknown
156	A/chicken/Egypt/N19598C/2021	III	Menia	Chicken	Healthy	Full genome	Farm	24/01/2021	Unknown
157	A/chicken/Egypt/N19598D/2021	III	Menia	Chicken	Healthy	Full genome	Farm	24/01/2021	Unknown
158	A/chicken/Egypt/N19599A/2021	III	Menia	Chicken	Healthy	Full genome	Farm	24/01/2021	Unknown
159	A/chicken/Egypt/N19600A/2021	III	Menia	Chicken	Healthy	Full genome	Farm	24/01/2021	Unknown
160	A/chicken/Egypt/N19600C/2021	III	Menia	Chicken	Healthy	Full genome	Farm	24/01/2021	Unknown
161	A/chicken/Egypt/N19600D/2021	III	Menia	Chicken	Healthy	Full genome	Farm	24/01/2021	Unknown
162	A/chicken/Egypt/N19604C/2021	III	Menia	Chicken	Healthy	Full genome	Farm	24/01/2021	Unknown
163	A/chicken/Egypt/N19604D/2021	III	Menia	Chicken	Healthy	Full genome	Farm	24/01/2021	Unknown
164	A/chicken/Egypt/N19606C/2021	III	Menia	Chicken	Healthy	Full genome	House	24/01/2021	Unknown
165	A/chicken/Egypt/A19610/2021	III	Assiut	Chicken	Healthy	Full genome	Market	26/01/2021	Unknown
166	A/chicken/Egypt/A19618/2021	III	Assiut	Chicken	Healthy	Full genome	Market	26/01/2021	Unknown
167	A/chicken/Egypt/A19669/2021	III	Assiut	Chicken	Healthy	Full genome	Market	10/02/2021	Unknown
168	A/chicken/Egypt/S19708B/2021	III	Sharqeia	Chicken	Healthy	Full genome	Farm	12/02/2021	Unknown
169	A/chicken/Egypt/S19712/2021	III	Sharqeia	Chicken	Healthy	Full genome	Market	12/02/2021	Unknown
170	A/chicken/Egypt/S19714/2021	III	Sharqeia	Chicken	Healthy	Full genome	Market	12/02/2021	Unknown
171	A/chicken/Egypt/N19764B/2021	III	Menia	Chicken	Healthy	Full genome	Farm	25/02/2021	Unknown
172	A/chicken/Egypt/N19764C/2021	III	Menia	Chicken	Healthy	Full genome	Farm	25/02/2021	Unknown
173	A/chicken/Egypt/N19766D/2021	III	Menia	Chicken	Healthy	Full genome	Farm	25/02/2021	Unknown

Table S2. Summary of the Egyptian AIV H9N2 genes analyzed in the current study with their accession numbers in GenBank.

H9N2 isolate	PB2	PB1	PA	HA	NP	NA	M	NS
A/chicken/Egypt/N13851C/2017	ON374814	ON374815	ON374816	ON374817	ON374818	ON399187	ON374819	ON374820
A/chicken/Egypt/F14014E/2017	ON374670	ON374671	ON374672	ON374673	ON374674	ON374675	ON374676	ON374677
A/chicken/Egypt/S14165/2017	ON374646	ON374647	ON374648	ON374649	ON374650	ON374651	ON374652	ON374653
A/chicken/Egypt/S14187B/2017	ON374774	ON374775	ON374776	ON374777	ON374778	ON374779	ON374780	ON374781
A/chicken/Egypt/Q14260A/2017	ON374574	ON374575	ON374576	ON374577	ON374578	ON374579	ON374580	ON374581

A/chicken/Egypt/N14498A/2017	ON374896	ON374897	ON374898	ON374899	ON374900	ON374901	ON374902	ON374903
A/chicken/Egypt/N14499B/2017	ON374526	ON374527	ON374528	ON374529	ON374530	ON374531	ON374532	ON374533
A/chicken/Egypt/N14500A/2017	ON374518	ON374519	ON374520	ON374521	ON374522	ON374523	ON374524	ON374525
A/chicken/Egypt/A14733/2017	ON374798	ON374799	ON374800	ON374801	ON374802	ON374803	ON374804	ON374805
A/chicken/Egypt/N14782D/2017	ON374726	ON374727	ON374728	ON374729	ON374730	ON374731	ON374732	ON374733
A/pigeon/Egypt/D14794/2017	ON374904	ON374905	ON374906	ON374907	ON374908	ON374909	ON374910	ON374911
A/chicken/EGYPT/A15043/2018	ON374742	ON374743	ON374744	ON374745	ON374746	ON374747	ON374748	ON374749
A/chicken/EGYPT/A15066/2018	ON373992	ON373993	ON373994	ON373995	ON373996	ON373997	ON373998	ON373999
A/chicken/EGYPT/A15068/2018	ON373979	ON373980	ON373981	MN038183	ON373982	ON373983	ON373984	ON373985
A/chicken/EGYPT/A15073/2018	ON411600	ON374912	ON374913	ON374914	ON374915	ON374916	ON374917	ON374918
A/chicken/EGYPT/A15074/2018	ON374882	ON374883	ON374884	MN038193	ON374885	ON374886	ON374887	ON374888
A/chicken/EGYPT/A15076/2018	ON374718	ON374719	ON374720	ON374721	ON374722	ON374723	ON374724	ON374725
A/chicken/EGYPT/A15333/2018	ON374875	ON374876	ON393926	ON374877	ON374878	ON374879	ON374880	ON374881
A/chicken/EGYPT/S15516/2018	ON374582	ON374583	ON374584	ON374585	ON374586	ON374587	ON374588	ON374589
A/chicken/EGYPT/S15518/2018	ON374455	ON374456	ON374457	ON374458	ON374459	ON374460	ON374461	ON374462
A/chicken/EGYPT/S15530C/2018	ON374598	ON374599	ON374600	ON374601	ON374602	ON374603	ON374604	ON374605
A/chicken/EGYPT/A15553C/2018	ON374889	ON374890	ON374891	ON374892	ON374893	ON381763	ON374894	ON374895
A/chicken/EGYPT/A15573/2018	ON374867	ON374868	ON374869	ON374870	ON374871	ON374872	ON374873	ON374874
A/chicken/EGYPT/A15582/2018	ON374008	ON374009	ON374010	ON374011	ON374012	ON374013	ON374014	ON374015
A/chicken/EGYPT/A15659/2018	ON374016	ON374017	ON374018	ON374019	ON374020	ON374021	ON374022	ON374023
A/chicken/EGYPT/A15660/2018	ON374000	ON374001	ON374002	ON374003	ON374004	ON374005	ON374006	ON374007
A/chicken/EGYPT/A15669/2018	ON374806	ON374807	ON374808	ON374809	ON374810	ON374811	ON374812	ON374813
A/chicken/EGYPT/A15799/2018	ON374734	ON374735	ON374736	ON374737	ON374738	ON374739	ON374740	ON374741
A/chicken/EGYPT/A15801/2018	ON374558	ON374559	ON374560	ON374561	ON374562	ON374563	ON374564	ON374565
A/chicken/EGYPT/A15802/2018	ON374851	ON374852	ON374853	ON374854	ON374855	ON374856	ON374857	ON374858
A/chicken/EGYPT/A15803/2018	ON374542	ON374543	ON374544	ON374545	ON374546	ON374547	ON374548	ON374549
A/chicken/EGYPT/D15830D/2018	ON374790	ON374791	ON374792	ON374793	ON374794	ON374795	ON374796	ON374797
A/chicken/EGYPT/N15888/2018	ON374859	ON374860	ON374861	ON374862	ON374863	ON374864	ON374865	ON374866
A/chicken/Egypt/S16688C/2019	MT261502	MT261498	MT261499	MT261496	MT261500	MT261495	MT261497	MT261501
A/chicken/Egypt/S16692/2019	MT261584	MT261586	MT261589	MT261585	MT261587	MT261591	MT261588	MT261590

A/chicken/Egypt/S16693/2019	MT261482	MT261483	MT261480	MT261481	MT261484	MT261479	MT261485	MT261486
A/chicken/Egypt/S16694/2019	MW137748	MW137746	MW137750	MW137752	MW137751	MW137749	ON399109	MW137747
A/chicken/Egypt/S16695/2019	MT261513	MT261515	MT261512	MT261518	MT261516	MT261514	MT261517	MT261511
A/chicken/Egypt/S16700B/2019	MT261390	MT261388	MT261387	MT261386	MT261392	MT261391	MT261389	MT261393
A/chicken/Egypt/S16700D/2019	MT261574	MT261569	MT261573	MT261571	MT261568	MT261572	MT261570	MT261567
A/chicken/Egypt/S16701B/2019	MT261537	MT261535	MT261542	MT261538	MT261536	MT261540	MT261539	MT261541
A/chicken/Egypt/S16702D/2019	MT261640	MT261636	MT261637	MT261639	MT261634	MT261638	MT261633	MT261635
A/chicken/Egypt/S16703B/2019	MT261700	MT261698	MT261701	MT261699	MT261704	MT261697	MT261702	MT261703
A/chicken/Egypt/S16703C/2019	MT261622	MT261624	MT261618	MT261623	MT261619	MT261620	MT261617	MT261621
A/chicken/Egypt/S16703D/2019	MT261681	MT261687	MT261685	MT261688	MT261684	MT261686	MT261682	MT261683
A/chicken/Egypt/S16704A/2019	MW137922	MW137924	MW137923	MW137763	MW137761	MW137760	MW137759	MW137762
A/chicken/Egypt/S16704B/2019	MW137897	MW137898	MW137903	MW137901	MW137896	MW137900	MW137899	MW137902
A/chicken/Egypt/S16704C/2019	MT261673	MT261677	MT261679	MT261675	MT261674	MT261680	MT261678	MT261676
A/chicken/Egypt/S16705A/2019	MT261564	MT261563	MT261560	MT261559	MT261561	MT261566	MT261565	MT261562
A/chicken/Egypt/S16705C/2019	MT261627	MT261626	MT261628	MT261631	MT261632	MT261625	MT261630	MT261629
A/chicken/Egypt/S16705D/2019	MT261604	MT261605	MT261607	MT261603	MT261602	MT261606	MT261601	MT261600
A/chicken/Egypt/N16735D/2019	MT261550	MT261543	MT261545	MT261548	MT261547	MT261546	MT261544	MT261549
A/chicken/Egypt/N16739A/2019	MT261466	MT261468	MT261463	MT261467	MT261465	MT261470	MT261469	MT261464
A/chicken/Egypt/F16772D/2019	MT261610	MT261614	MT261609	MT261611	MT261615	MT261608	MT261613	MT261612
A/chicken/Egypt/F16774A/2019	MT261648	MT261646	MT261643	MT261645	MT261644	MT261647	MT261642	MT261641
A/chicken/Egypt/F16774B/2019	MT261442	MT261445	MT261443	MT261438	MT261439	MT261440	MT261441	MT261444
A/chicken/Egypt/A16777/2019	MT261594	MT261599	MT261598	MT261597	MT261595	MT261593	MT261596	MT261592
A/chicken/Egypt/A16857/2019	MW137696	MW137698	MW137967	MW137966	MW137695	MW137694	MW137968	MW137697
A/chicken/Egypt/A16859/2019	MW137820	MW137823	MW137826	MW137821	MW137825	MW137822	MW137952	MW137824
A/chicken/Egypt/A16860/2019	MW137963	MW137991	MW137964	MW137965	MW137988	MW137990	MW137962	MW137989
A/chicken/Egypt/A16863/2019	MW137811	MW137807	MW137809	MW137992	MW137806	MW137808	MW137993	MW137810
A/pigeon/Egypt/A16865/2019	MW137955	MW137997	MW137956	MW137994	MW137996	MW137995	MW137953	MW137954
A/chicken/Egypt/A16886/2019	MW137958	MW137828	MW137827	MW137830	MW137829	MW137831	MW137957	MW137832
A/chicken/Egypt/N16941C/2019	MW137688	MW137687	MW137690	MW137689	MW137692	MW137691	MW137948	MW137693
A/chicken/Egypt/N16946C/2019	MT261488	MT261487	MT261493	MT261489	MT261492	MT261490	MT261494	MT261491

A/chicken/Egypt/S16965D/2019	MW137741	MW137738	MW137740	MW137734	MW137736	MW137735	MW137737	MW137739
A/chicken/Egypt/N17040A/2019	MW137939	MW137816	MW137940	MW137941	MW137817	MW137819	MW137938	MW137818
A/chicken/Egypt/N17040B/2019	MW137960	MW137755	MW137961	MW137754	MW137756	MW137753	MW137959	MW137757
A/chicken/Egypt/N17041A/2019	MW137909	MW137911	MW137910	MW137907	MW137908	MW137845	MW137906	MW137844
A/chicken/Egypt/N17041C/2019	MW596003	MW137859	MW137927	MW137926	MW137856	MW137858	MW137925	MW137857
A/chicken/Egypt/N17048C/2019	MW137918	MW137700	MW137920	MW137699	MW137703	MW137702	MW137919	MW137701
A/chicken/Egypt/N17056/2019	MW137722	MW137723	MW137568	MW137720	MW137725	MW137721	MW137569	MW137724
A/chicken/Egypt/F17123A/2019	MW137876	MW137877	MW137881	MW137882	MW137878	MW137875	MW137880	MW137879
A/chicken/Egypt/F17124B/2019	MW137893	MW137895	MW137928	MW137891	MW137892	MW137890	MW137929	MW137894
A/chicken/Egypt/F17124C/2019	MW137914	MW137915	MW137917	MW137916	MW137912	MW137904	MW137913	MW137905
A/chicken/Egypt/F17124D/2019	MW137987	MW137985	MW137983	MW137986	MW137984	MW137680	MW137982	MW137679
A/chicken/Egypt/F17133A/2019	MW138001	MW137932	MW138003	MW137999	MW138002	MW137930	MW138000	MW137931
A/chicken/Egypt/A17349/2019	MW138042	MW138040	MW138037	MW138036	MW138041	MW138038	MW138095	MW138039
A/chicken/Egypt/A17358/2019	MW137945	MW137947	MW137943	MW137944	MW137572	MW137946	MW137571	MW137942
A/chicken/Egypt/A17416/2019	MW138093	MW137980	MW138091	MW137978	MW137979	MW137977	MW138092	MW137981
A/chicken/Egypt/A17561/2019	MW137871	MW137872	MW137870	MW137873	MW137874	MW137868	MW137570	MW137869
A/chicken/Egypt/Q17812B/2019	MW137704	MW137711	MW137710	MW137709	MW137705	MW137706	MW137707	MW137708
A/chicken/Egypt/Q17897C/2019	MW137727	MW137732	MW137730	MW137729	MW137731	MW137733	MW137728	MW137726
A/chicken/Egypt/D17949A/2019	ON374758	ON374759	ON374760	ON374761	ON374762	ON374763	ON374764	ON374765
A/chicken/Egypt/D17949B/2019	MW137867	MW137861	MW137860	MW137865	MW137866	MW137864	MW137862	MW137863
A/chicken/Egypt/D17949C/2019	MW137788	MW137787	MW137785	MW137784	MW137790	MW137786	MW137791	MW137789
A/chicken/Egypt/A17969/2019	MW137971	MW137972	MW137970	MW137975	MW137969	MW137974	MW137973	MW137976
A/chicken/Egypt/S18034E/2019	MW137783	MW137776	MW137777	MW137781	MW137782	MW137778	MW137780	MW137779
A/chicken/Egypt/Q18036B/2019	MW137714	MW137713	MW137718	MW137719	MW137717	MW137715	MW137712	MW137716
A/chicken/Egypt/Q18036E/2019	MW137675	MW137673	MW137677	MW137676	MW137674	MW137671	MW137672	MW137678
A/chicken/Egypt/Q18041A/2019	MW137766	MW137767	MW137770	MW137768	MW137765	MW137771	MW137764	MW137769
A/pigeon/Egypt/A18091/2019	ON374622	ON374623	ON374624	ON374625	ON374626	ON374627	ON374628	ON374629
A/pigeon/Egypt/A18092/2019	ON374638	ON374639	ON374640	ON374641	ON374642	ON374643	ON374644	ON374645
A/pigeon/Egypt/A18093/2019	ON374843	ON374844	ON374845	ON374846	ON374847	ON374848	ON374849	ON374850

A/pigeon/Egypt/A18094/2019	ON374782	ON374783	ON374784	ON374785	ON374786	ON374787	ON374788	ON374789
A/chicken/Egypt/F18299B/2020	ON374662	ON374663	ON374664	ON374665	ON374666	ON374667	ON374668	ON374669
A/chicken/Egypt/F18299C/2020	ON374510	ON374511	ON374512	ON374513	ON374514	ON374515	ON374516	ON374517
A/chicken/Egypt/A18495/2020	ON374534	ON374535	ON374536	ON374537	ON374538	ON374539	ON374540	ON374541
A/chicken/Egypt/A18496/2020	ON374766	ON374767	ON374768	ON374769	ON374770	ON374771	ON374772	ON374773
A/chicken/Egypt/A18497/2020	ON374478	ON374479	ON374480	ON374481	ON374482	ON374483	ON374484	ON374485
A/chicken/Egypt/A18498/2020	ON374702	ON374703	ON374704	ON374705	ON374706	ON374707	ON374708	ON374709
A/chicken/Egypt/A18503/2020	ON374750	ON374751	ON374752	ON374753	ON374754	ON374755	ON374756	ON374757
A/chicken/Egypt/A18504/2020	ON374919	ON374920	ON374921	ON374922	ON374923	ON374924	ON374925	ON374926
A/chicken/Egypt/A18505/2020	ON374606	ON374607	ON374608	ON374609	ON374610	ON374611	ON374612	ON374613
A/chicken/Egypt/A18507/2020	ON374502	ON374503	ON374504	ON374505	ON374506	ON374507	ON374508	ON374509
A/chicken/Egypt/S18523C/2020	ON374710	ON374711	ON374712	ON374713	ON374714	ON374715	ON374716	ON374717
A/chicken/Egypt/S18527/2020	ON374550	ON374551	ON374552	ON374553	ON374554	ON374555	ON374556	ON374557
A/chicken/Egypt/D18579A/2020	ON374694	ON374695	ON374696	ON374697	ON374698	ON374699	ON374700	ON374701
A/chicken/Egypt/D18592/2020	ON374590	ON374591	ON374592	ON374593	ON374594	ON374595	ON374596	ON374597
A/chicken/Egypt/A18605/2020	ON374686	ON374687	ON374688	ON374689	ON374690	ON374691	ON374692	ON374693
A/chicken/Egypt/A18607/2020	ON374566	ON374567	ON374568	ON374569	ON374570	ON374571	ON374572	ON374573
A/chicken/Egypt/A18610/2020	ON374678	ON374679	ON374680	ON374681	ON374682	ON374683	ON374684	ON374685
A/chicken/Egypt/S18643C/2020	ON374614	ON374615	ON374616	ON374617	ON374618	ON374619	ON374620	ON374621
A/chicken/Egypt/S18643D/2020	ON374486	ON374487	ON374488	ON374489	ON374490	ON374491	ON374492	ON374493
A/chicken/Egypt/A18716/2020	ON374494	ON374495	ON374496	ON374497	ON374498	ON374499	ON374500	ON374501
A/chicken/Egypt/A18717/2020	ON374630	ON374631	ON374632	ON374633	ON374634	ON374635	ON374636	ON374637
A/chicken/Egypt/A18720/2020	ON374654	ON374655	ON374656	ON374657	ON374658	ON374659	ON374660	ON374661
A/chicken/Egypt/S18755A/2020	ON374078	ON374079	ON374080	ON374081	ON374082	ON374083	ON374084	ON374085
A/chicken/Egypt/S18755B/2020	ON374187	ON374188	ON374189	ON374190	ON374191	ON374192	ON374193	ON374194
A/chicken/Egypt/S18755C/2020	ON374283	ON374284	ON374285	ON374286	ON374287	ON374288	ON374289	ON374290
A/chicken/Egypt/S18755D/2020	ON381746	ON381747	ON381748	ON381749	ON374067	ON381750	ON374068	ON374069
A/chicken/Egypt/N18851C/2020	ON374375	ON374376	ON374377	ON374378	ON374379	ON374380	ON374381	ON374382
A/chicken/Egypt/S18985A/2020	ON374219	ON374220	ON374221	ON374222	ON374223	ON374224	ON374225	ON374226
A/chicken/Egypt/S18985B/2020	ON374235	ON374236	ON374237	ON374238	ON374239	ON374240	ON374241	ON374242

A/chicken/Egypt/S18992/2020	ON374027	ON374028	ON374029	ON374030	ON374031	ON374032	ON374033	ON374034
A/chicken/Egypt/N19072B/2020	ON374398	ON374399	ON374400	ON374401	ON374402	ON374403	ON374404	ON374405
A/chicken/Egypt/N19072C/2020	ON374323	ON374324	ON374325	ON374326	ON374327	ON374328	ON374329	ON374330
A/chicken/Egypt/N19074A/2020	ON381751	ON381752	ON381753	ON374371	ON374372	ON381754	ON374373	ON374374
A/chicken/Egypt/N19078B/2020	ON374251	ON374252	ON374253	ON374254	ON374255	ON374256	ON374257	ON374258
A/chicken/Egypt/A19165/2020	ON374059	ON374060	ON374061	ON374062	ON374063	ON374064	ON374065	ON374066
A/chicken/Egypt/A19244/2020	ON374347	ON374348	ON374349	ON374350	ON374351	ON374352	ON374353	ON374354
A/chicken/Egypt/A19245/2020	ON374406	ON374407	ON374408	ON374409	ON374410	ON374411	ON374412	ON374413
A/chicken/Egypt/A19246/2020	ON374436	ON374437	ON374438	ON374439	ON374440	ON374441	ON374442	ON374443
A/duck/Egypt/D19288B/2020	ON381761	ON381762	ON374430	ON374431	ON374432	ON374433	ON374434	ON374435
A/chicken/Egypt/D19290A/2020	ON374414	ON374415	ON374416	ON374417	ON374418	ON374419	ON374420	ON374421
A/chicken/Egypt/D19290B/2020	ON381756	ON381757	ON381758	ON381759	ON374444	ON381760	ON374445	ON374446
A/chicken/Egypt/D19290C/2020	ON381755	ON374463	ON374464	ON374465	ON374466	ON374467	ON374468	ON374469
A/chicken/Egypt/D19290D/2020	ON374355	ON374356	ON374357	ON374358	ON374359	ON374360	ON374361	ON374362
A/chicken/Egypt/D19292/2020	ON374363	ON374364	ON374365	ON374366	ON374367	ON374368	ON374369	ON374370
A/chicken/Egypt/D19293/2020	ON374470	ON374471	ON374472	ON374473	ON374474	ON374475	ON374476	ON374477
A/chicken/Egypt/N19302B/2020	ON374291	ON374292	ON374293	ON374294	ON374295	ON374296	ON374297	ON374298
A/chicken/Egypt/N19304A/2020	ON374147	ON374148	ON374149	ON374150	ON374151	ON374152	ON374153	ON374154
A/chicken/Egypt/S19326A/2020	ON374123	ON374124	ON374125	ON374126	ON374127	ON374128	ON374129	ON374130
A/chicken/Egypt/S19326B/2020	ON374107	ON374108	ON374109	ON374110	ON374111	ON374112	ON374113	ON374114
A/chicken/Egypt/S19326C/2020	ON374275	ON374276	ON374277	ON374278	ON374279	ON374280	ON374281	ON374282
A/chicken/Egypt/S19326D/2020	ON374195	ON374196	ON374197	ON374198	ON374199	ON374200	ON374201	ON374202
A/chicken/Egypt/S19338A/2020	ON374179	ON374180	ON374181	ON374182	ON374183	ON374184	ON374185	ON374186
A/chicken/Egypt/S19338B/2020	ON374051	ON374052	ON374053	ON374054	ON374055	ON374056	ON374057	ON374058
A/chicken/Egypt/S19338D/2020	ON374070	ON374071	ON374072	ON374073	ON374074	ON374075	ON374076	ON374077
A/pigeon/Egypt/D19471/2020	ON374163	ON374164	ON374165	ON374166	ON374167	ON374168	ON374169	ON374170
A/chicken/Egypt/D19479/2020	ON374099	ON374100	ON374101	ON374102	ON374103	ON374104	ON374105	ON374106
A/chicken/Egypt/D19481/2020	ON374139	ON374140	ON374141	ON374142	ON374143	ON374144	ON374145	ON374146
A/chicken/Egypt/D19486A/2020	ON374171	ON374172	ON374173	ON374174	ON374175	ON374176	ON374177	ON374178
A/chicken/Egypt/D19486B/2020	ON374299	ON374300	ON374301	ON374302	ON374303	ON374304	ON374305	ON374306

A/chicken/Egypt/N19598A/2021	ON374331	ON374332	ON374333	ON374334	ON374335	ON374336	ON374337	ON374338
A/chicken/Egypt/N19598C/2021	ON374307	ON374308	ON374309	ON374310	ON374311	ON374312	ON374313	ON374314
A/chicken/Egypt/N19598D/2021	ON374043	ON374044	ON374045	ON374046	ON374047	ON374048	ON374049	ON374050
A/chicken/Egypt/N19599A/2021	ON374115	ON374116	ON374117	ON374118	ON374119	ON374120	ON374121	ON374122
A/chicken/Egypt/N19600A/2021	ON374390	ON374391	ON374392	ON374393	ON374394	ON374395	ON374396	ON374397
A/chicken/Egypt/N19600C/2021	ON374383	ON374384	ON374385	ON399186	ON374386	ON374387	ON374388	ON374089
A/chicken/Egypt/N19600D/2021	ON374203	ON374204	ON374205	ON374206	ON374207	ON374208	ON374209	ON374210
A/chicken/Egypt/N19604C/2021	ON374267	ON374268	ON374269	ON374270	ON374271	ON374272	ON374273	ON374274
A/chicken/Egypt/N19604D/2021	ON374315	ON374316	ON374317	ON374318	ON374319	ON374320	ON374321	ON374322
A/chicken/Egypt/N19606C/2021	ON374259	ON374260	ON374261	ON374262	ON374263	ON374264	ON374265	ON374266
A/chicken/Egypt/A19610/2021	ON374422	ON374423	ON374424	ON374425	ON374426	ON374427	ON374428	ON374429
A/chicken/Egypt/A19618/2021	ON374339	ON374340	ON374341	ON374342	ON374343	ON374344	ON374345	ON374346
A/chicken/Egypt/A19669/2021	ON374447	ON374448	ON374449	ON374450	ON374451	ON374452	ON374453	ON374454
A/chicken/Egypt/S19708B/2021	ON374243	ON374244	ON374245	ON374246	ON374247	ON374248	ON374249	ON374250
A/chicken/Egypt/S19712/2021	ON374035	ON374036	ON374037	ON374038	ON374039	ON374040	ON374041	ON374042
A/chicken/Egypt/S19714/2021	ON374155	ON374156	ON374157	ON374158	ON374159	ON374160	ON374161	ON374162
A/chicken/Egypt/N19764B/2021	ON374211	ON374212	ON374213	ON374214	ON374215	ON374216	ON374217	ON374218
A/chicken/Egypt/N19764C/2021	ON374227	ON374228	ON374229	ON374230	ON374231	ON374232	ON374233	ON374234
A/chicken/Egypt/N19766D/2021	ON374131	ON374132	ON374133	ON374134	ON374135	ON374136	ON374137	ON374138

Table S3. Analysis of genetic markers associated with host range specificity in the PB2, PB1-F2, PB1, PA, NP, M1, M2 and NS1 proteins in 173 H9N2 viruses isolated from poultry in Egypt. The avian or mammalian-preference markers are shown.

Viral Protein	aa Site	Avian Preference	Mammalian Preference	Subtypes	Egyptian H9N2 (2017-2021) =173	Reference
PB2	44	A	S	(H5N1, H9N2, H7N2, H7N7), (H5N1, H9N2)	A (155), N/A (18)	[1,2]
	64	M	T	H5N1	M (84), T (65), K (5), N/A (19)	[3]
	81	T	M	H5N1, H9N2	T (141), I (14), N/A (18)	[2]
	199	A	S	(H5N1, H9N2, H7N2, H7N7), (H5N1, H9N2)	A (154), S (1), N/A (18)	[1,2]
	318	K	R	H3N2	R (160), N/A (13)	[4]
	627	E	K	H9N2	E (172), K (1)	[5]
	661	A	T	H1N1	A (162), V (10), T (1)	[6]
	701	D	N	H1N1	D (173)	[7]
	702	K	R	H1N1	K (173)	[6]
PB1	336	V	I	H5N1, H9N2, H7N2, H7N7	V (170), N/A (3)	[1]
	375	N	S	H1N1	N (165), T (3), S (3), H (1), N/A (1)	[8]
PA	28	P	L	H5N1, H9N2	P (155), N/A (18)	[9]

	55	D	N	(H5N1, H9N2, H7N2, H7N7), (H5N1, H9N2)	D (155), N/A (18)	[1,2]
	57	R	Q	H5N1, H9N2, H7N2, H7N7	R (141), Q (14), N/A (18)	[1]
	100	V	A	H7N9	V (142), A (13), N/A (18)	[10]
	133	E	G	H3N2	E (155), N/A (18)	[11]
	225	S	C	H1N1, H2H2, H3N2, H5N1	S (155), N (1), N/A (17)	[12]
	241	C	Y	H5N1	C (156), N/A (17)	[13]
	268	L	I	H1N1, H2H2, H3N2, H5N1	L (156), I (1), N/A (16)	[12]
	312	K	R	H5N1, H9N2	K (163), N/A (10)	[2]
	356	K	R	H5N1, H9N2, H7N2, H7N7	K (173)	[1]
	382	E	D	(H5N1, H9N2), H1N1	E (18), D (155)	[2,8]
	404	A	S	H5N1, H9N2, H7N2, H7N7	A (173)	[1]
	409	S	N	(H5N1, H9N2, H7N2, H7N7), (H5N1, H9N2)	S (167), N (6)	[1,2]
	552	T	S	H1N1, H2H2, H3N2, H5N1	T (173)	[12]
	615	K	L	H7N7	K (173)	[14]
NP	31	R	K	H5N1, H9N2	R (168), N/A (5)	[2]
	33	V	I	(H5N1, H9N2, H7N2, H7N7), (H5N1, H9N2)	V (165), I (3), N/A (5)	[1,2]
	61	I	L	(H1N1, H2H2, H3N2, H5N1), (H5N1, H9N2)	I (161), L (7), N/A (5)	[2,12]
	100	R	V	(H5N1, H9N2, H7N2, H7N7), (H5N1, H9N2)	R (168), N/A (5)	[1,2]
	109	I	V	H5N1, H9N2, H7N2, H7N7	I (131), T (37), N/A (5)	[1]
	127	E	D	H5N1, H9N2	E (168), N/A (5)	[2]
	136	L	M	H5N1, H9N2	L (168), N/A (5)	[2]
	214	R	K	(H5N1, H9N2, H7N2, H7N7), (H5N1, H9N2)	K (171), R (1), N/A (1)	[1,2]
	283	L	P	(H5N1, H9N2, H7N2, H7N7), (H5N1, H9N2)	L (173)	[1,2]
	293	R	K	(H5N1, H9N2, H7N2, H7N7), (H5N1, H9N2)	R (173)	[1,2]
	305	R	K	H5N1, H9N2, H7N2, H7N7	R (173)	[1]
	313	F	Y	(H5N1, H9N2, H7N2, H7N7), (H5N1, H9N2)	F (173)	[1,2]
	357	Q	K	H5N1, H9N2, H7N2, H7N7	Q (171), R (2)	[1]
	372	E	D	H5N1, H9N2, H7N2, H7N7	E (173)	[1]
	375	D	G/E	H5N1, H9N2	D (171), E (2)	[2]
	422	R	K	H5N1, H9N2, H7N2, H7N7	R (173)	[1]
	442	T	A	H5N1, H9N2, H7N2, H7N7	T (173)	[1]
	455	D	E	H5N1, H9N2, H7N2, H7N7	D (173)	[1]
M1	15	V	I	H5N1	I (151), N/A (22)	[15]
	115	V	I	H1N1, H2H2, H3N2, H5N1	V (152), N/A (21)	[12]
	121	T	A	H1N1, H2H2, H3N2, H5N1	T (153), N/A (20)	[12]
	137	T	A	(H1N1, H2H2, H3N2, H5N1), (H5N1, H9N2)	T (153), N/A (20)	[2,12]
M2	11	T	I	H5N1, H9N2, H7N2, H7N7	T (173)	[1]
	16	E	G/D	H5N1, H9N2	G (172), V (1)	[2]
	20	S	N	(H5N1, H9N2, H7N2, H7N7), (H5N1, H9N2)	S (162), N (11)	[1,2]
	28	I	I/V	H5N1, H9N2	V (158), I (15)	[2]
	57	Y	H	H5N1, H9N2, H7N2, H7N7	Y (173)	[1]
	55	L	F	H1N1	F (170), L (3)	[16]
	86	V	A	H5N1, H9N2, H7N2, H7N7	V (173)	[1]
NS1	227	E	K/R	H7N1	E (151), G (16), N/A (6)	[17]

Table S4. Comparison of the Glycosylation sites, RBS, and antigenic sites of the HA of H9N2 viruses isolated from poultry in Egypt between 2017 and 2021.

	**Glycosylation Sites**									**RBS**								**Antigenic site I (A)**			**Antigenic site II (B)**			**Overlapping site**		
**H9N2 Viruses (H9 numbering)**	29	105	141	196	206	218	298	305	492	166	191	197	198	232	234	235	236	143T	166 N	170P	153 D	201 N	234L	141 N	197T	206T
A/quail/Hong Kong/G1/97	**NST**	**NGT**	**NVT**	**YTE**	**NDT**	**NRT**	**NST**	**NIS**	**NGT**	**S**	**H**	**T**	**E**	**N**	**L**	**Q**	**G**									
A/duck/Hong Kong/Y280/97	**NST**	**NGL**	**NVS**	**DTT**	**TDT**	**NRT**	**NTT**	**NVS**	**NGT**	**N**	**N**	**T**	**T**	**N**	**L**	**Q**	**G**									
A/quail/Egypt/D9842/2014	**NST**	**NGT**	**NVT**	**DTT**	**TDT**	**DRT**	**NST**	**NIS**	**NGT**	**N**	**N**	**T**	**T**	**N**	**L**	**I**	**G**									
A/quail/Egypt/D10093/2014	**NST**	**NGT**	**NVT**	**NTT**	**TDT**	**DRT**	**NST**	**NIS**	**NGT**	**D**	**H**	**T**	**T**	**N**	**L**	**T**	**G**	**T**	**D**	**P**	**N**	**A**	**L**	**N**	**T**	**T**
A/quail/Egypt/D10105/2014	**NST**	**NGT**	**NVT**	**NTT**	**TDT**	**DRT**	**NST**	**NIS**	**NGT**	**D**	**H**	**T**	**T**	**N**	**L**	**T**	**G**	**T**	**D**	**P**	**N**	**A**	**L**	**N**	**T**	**T**
A/quail/Egypt/D10106/2014	**NST**	**NGT**	**NVT**	**NTT**	**TDT**	**DRT**	**NST**	**NIS**	**NGT**	**D**	**H**	**T**	**T**	**N**	**L**	**T**	**G**	**T**	**D**	**P**	**N**	**A**	**L**	**N**	**T**	**T**
A/chicken/Egypt/A14733/2017	NST	NGT	NVT	DTA	TDT	DRT	NST	NIS	NGT	N	H	T	A	N	L	I	G	T	N	P	D	N	L	N	T	T
A/pigeon/Egypt/D14794/2017	NST	NGT	NVT	DTT	TDT	DRT	NST	NIS	NGT	N	H	T	T	N	L	I	G	T	N	P	D	N	L	N	T	T
A/chicken/Egypt/F14014E/2017	NST	NGT	NVT	DTT	TDT	DRT	NST	NIS	NGT	N	H	T	T	N	L	I	G	T	N	P	D	N	L	N	T	T
A/chicken/Egypt/N13851C/2017	NST	NGT	NVT	DTT	TDT	DRT	NST	NIS	NGT	N	H	T	T	N	L	I	G	T	N	P	D	D	L	N	T	T
A/chicken/Egypt/N14498A/2017	NST	NGT	NVT	DTT	TDT	DRT	NST	NIS	NGT	N	H	T	T	N	L	I	G	T	N	P	D	N	L	N	T	T
A/chicken/Egypt/N14499B/2017	NST	NGT	NVT	DTT	TDT	DRT	NST	NIS	NGT	N	H	T	T	N	L	I	G	T	N	P	D	N	L	N	T	T
A/chicken/Egypt/N14500A/2017	NST	NGT	NVT	DTT	TDT	DRT	NST	NIS	NGT	N	H	T	T	N	L	I	G	T	N	P	D	N	L	N	T	T
A/chicken/Egypt/N14782D/2017	NST	NGT	NVT	DTA	TDT	DRT	NST	NIS	NGT	N	H	T	A	N	L	I	G	T	N	P	D	N	L	N	T	T
A/chicken/Egypt/Q14260A/2017	NST	NGT	NVT	DTT	TDT	DRT	NST	NIS	NGT	N	H	T	T	N	L	I	G	T	N	P	D	N	L	N	T	T
A/chicken/Egypt/S14165/2017	NST	NGT	NVT	DTV	TDT	DRT	NST	NIS	NGT	N	H	T	V	N	L	I	G	T	N	P	D	N	L	N	T	T
A/chicken/Egypt/S14187B/2017	NST	NGT	NVT	DTV	TDT	DRT	NST	NIS	NGT	N	H	T	V	N	L	I	G	T	N	P	D	N	L	N	T	T
A/CHICKEN/EGYPT/A15043/2018	NST	NGT	NVT	DTT	TDT	DRT	NST	NVS	NGT	N	H	T	T	N	L	I	G	T	N	P	D	N	L	N	T	T
A/CHICKEN/EGYPT/A15068/2018	NST	NGT	NVT	DTA	TDT	DRT	NST	NIS	NGT	N	H	T	A	N	L	I	G	T	N	P	D	N	L	N	T	T
A/CHICKEN/EGYPT/A15073/2018	NST	NGT	NVT	DTA	TDT	DRT	NST	NVS	NGT	N	H	T	A	N	L	I	G	T	N	P	D	N	L	N	T	T
A/CHICKEN/EGYPT/A15074/2018	NST	NGT	NVT	DTA	TDT	DRT	NST	NVS	NGT	N	H	T	A	N	L	I	G	T	N	P	D	N	L	N	T	T
A/CHICKEN/EGYPT/A15076/2018	NST	NGT	NVT	DTA	TDT	DRT	NST	NVS	NGT	N	H	T	A	N	L	I	G	T	N	P	D	N	L	N	T	T
A/CHICKEN/EGYPT/A15333/2018	NST	NGT	NVT	DTA	TDT	DRT	NST	NIS	NGT	N	H	T	A	N	L	I	G	T	N	P	D	N	L	N	T	T
A/CHICKEN/EGYPT/A15553C/2018	NST	NGT	NVT	DTT	TDT	DRT	NST	NIS	NGT	N	H	T	T	N	L	I	G	T	N	P	D	N	L	N	T	T
A/CHICKEN/EGYPT/A15573/2018	NST	NGT	NVT	DTA	TDT	DRT	NST	NIS	NGT	N	H	T	A	N	L	I	G	T	N	P	D	N	L	N	T	T
A/CHICKEN/EGYPT/A15582/2018	NST	NGT	NVT	DTV	TDT	DRT	NST	NIS	NGT	N	H	T	V	N	L	I	G	T	N	P	D	N	L	N	T	T
A/CHICKEN/EGYPT/A15659/2018	NST	NGT	NVT	DTV	TDT	DRT	NST	NIS	NGT	S	H	T	V	N	L	I	G	T	S	P	D	N	L	N	T	T

A/CHICKEN/EGYPT/A15660/2018	NST	NGT	NVT	DTA	TDT	DRT	NST	NIS	NGT	S	H	T	A	N	L	I	G	T	S	P	D	N	L	N	T	T
A/CHICKEN/EGYPT/A15669/2018	NST	NGT	NVT	DTA	TDT	DRT	NST	NIS	NGT	S	H	T	A	N	L	I	G	T	S	P	D	N	L	N	T	T
A/CHICKEN/EGYPT/A15799/2018	NST	NGT	NVT	DTV	TDT	DRT	NST	NIS	NGT	N	H	T	V	N	L	I	G	T	N	P	G	N	L	N	T	T
A/CHICKEN/EGYPT/A15801/2018	NST	NGT	NVT	DTV	TDT	DRT	NST	NIS	NGT	N	H	T	V	N	L	I	G	T	N	P	G	N	L	N	T	T
A/CHICKEN/EGYPT/A15802/2018	NST	NGT	NVT	DTV	TDT	DRT	NST	NIS	NGT	N	H	T	V	N	L	I	G	T	N	P	G	N	L	N	T	T
A/CHICKEN/EGYPT/A15803/2018	NST	NGT	NVT	DTV	TDT	DRT	NST	NIS	NGT	N	H	T	V	N	L	I	G	T	N	P	G	N	L	N	T	T
A/CHICKEN/EGYPT/D15830D/2018	NST	NGT	NVT	DTA	TDT	DRT	NST	NIS	NGT	N	H	T	A	N	L	I	G	T	N	P	D	N	L	N	T	T
A/CHICKEN/EGYPT/N15888/2018	NST	NGT	NVT	DTA	TDT	DRT	NST	NIS	NGT	N	H	T	A	N	L	I	G	T	N	P	D	N	L	N	T	T
A/CHICKEN/EGYPT/S15516/2018	NST	NGT	NVT	DTT	TDT	DRT	NST	NIS	NGT	N	H	T	T	N	L	I	G	T	N	P	D	N	L	N	T	T
A/CHICKEN/EGYPT/S15518/2018	NST	NGT	NVT	DTT	TDT	DRT	NST	NIS	NGT	N	H	T	T	N	L	I	G	T	N	P	D	N	L	N	T	T
A/CHICKEN/EGYPT/S15530C/2018	NST	NGT	NVT	DTT	TDT	DRT	NST	NIS	NGT	N	H	T	T	N	L	I	G	T	N	P	D	N	L	N	T	T
A/CHICKEN/EGYPT/A15066/2018	NST	NGT	NVT	DTA	TDT	DRT	NST	NIS	NGT	N	H	T	A	N	L	I	G	T	N	P	D	N	L	N	T	T
A/chicken/Egypt/S16702D/2019	NST	NGT	NVT	DTA	TDT	DRT	NST	NIS	NGT	N	H	T	A	N	L	I	G	T	N	P	D	N	L	N	T	T
A/chicken/Egypt/S16703C/2019	NST	NGT	NVT	DTA	TDT	DRT	NST	NIS	NGT	N	H	T	A	N	L	I	G	T	N	P	D	N	L	N	T	T
A/chicken/Egypt/S16703D/2019	NST	NGT	NVT	DTA	TDT	DRT	NST	NIS	NGT	N	H	T	A	N	L	I	G	T	N	P	D	N	L	N	T	T
A/chicken/Egypt/S16704A/2019	NST	NGT	NVT	DTA	TDT	DRT	NST	NIS	NGT	N	H	T	A	N	L	I	G	T	N	P	D	N	L	N	T	T
A/chicken/Egypt/S16704B/2019	NST	NGT	NVT	DTA	TDT	DRT	NST	NIS	NGT	N	H	T	A	N	L	I	G	T	N	P	D	N	L	N	T	T
A/chicken/Egypt/S16704C/2019	NST	NGT	NVT	DTA	TDT	DRT	NST	NIS	NGT	N	H	T	A	N	L	I	G	T	N	P	D	N	L	N	T	T
A/chicken/Egypt/S16705A/2019	NST	NGT	NVT	DTA	TDT	DRT	NST	NIS	NGT	N	H	T	A	N	L	I	G	T	N	P	D	N	L	N	T	T
A/chicken/Egypt/S16705C/2019	NST	NGT	NVT	DTA	TDT	DRT	NST	NIS	NGT	N	H	T	A	N	L	I	G	T	N	P	D	N	L	N	T	T
A/chicken/Egypt/S16705D/2019	NST	NGT	NVT	DTA	TDT	DRT	NST	NIS	NGT	N	H	T	A	N	L	I	G	T	N	P	D	N	L	N	T	T
A/chicken/Egypt/N16735D/2019	NST	NGT	NVT	DTA	TDT	DRT	NST	NIS	NGT	N	H	T	A	N	L	I	G	T	N	P	D	N	L	N	T	T
A/chicken/Egypt/N16739A/2019	NST	NGT	NVT	DTA	TDT	DRT	NST	NIS	NGT	N	H	T	A	N	L	I	G	T	N	P	D	N	L	N	T	T
A/chicken/Egypt/F16772D/2019	NST	NGT	NVT	DTV	TDT	DRT	NST	NIS	NGT	N	H	T	V	N	L	I	G	T	N	P	D	N	L	N	T	T
A/chicken/Egypt/F16774A/2019	NST	NGT	NVT	DTA	TDT	DRT	NST	NIS	NGT	N	H	T	A	N	L	I	G	T	N	P	D	N	L	N	T	T
A/chicken/Egypt/F16774B/2019	NST	NGT	NVT	DTA	TDT	DRT	NST	NIS	NGT	N	H	T	A	N	L	I	G	T	N	P	D	N	L	N	T	T
A/chicken/Egypt/A16777/2019	NST	NGT	NVT	DTA	TDT	DRT	NST	NIS	NGT	N	H	T	A	N	L	I	G	T	N	P	D	N	L	N	T	T
A/chicken/Egypt/A16857/2019	NST	NGT	NVT	---	---	---	---	---	---	N	-	-	-	-	-	-	-	T	N	P	D	-	-	N	-	-
A/chicken/Egypt/A16859/2019	NST	NGT	NVT	DTT	TDT	DRT	NST	NIS	NGT	N	H	T	T	N	L	I	G	T	N	P	D	N	L	N	T	T
A/chicken/Egypt/A16860/2019	---	---	---	---	---	---	---	---	NGT	-	-	-	-	-	-	-	-	-	-	-	-	-	-	-	-	-
A/chicken/Egypt/A16863/2019	---	---	---	---	---	---	---	---	NGT	-	-	-	-	-	-	-	-	-	-	-	-	-	-	-	-	-

A/pigeon/Egypt/A16865/2019	NST	NGT	NVT	DTA	TDT	DRT	NST	NIS	NGT	N	H	T	A	N	L	I	G	T	N	P	D	N	L	N	T	T
A/chicken/Egypt/A16886/2019	NST	NGT	NVT	DTA	TDT	DRT	NST	NIS	NGT	N	H	T	A	N	L	I	G	T	N	P	D	N	L	N	T	T
A/chicken/Egypt/N16941C/2019	NST	NGT	NVT	DTA	TDT	DRT	NST	NVS	NGT	N	H	T	A	N	L	I	G	T	N	P	D	N	L	N	T	T
A/chicken/Egypt/N16946C/2019	NST	NGT	NVT	DTA	TDT	DRT	NST	NVS	NGT	N	H	T	A	N	L	I	G	T	N	P	D	N	L	N	T	T
A/chicken/Egypt/S16965D/2019	NST	NGT	NVT	DTA	TDT	DRT	NST	NIS	NGT	N	H	T	A	N	L	I	G	T	N	P	D	N	L	N	T	T
A/chicken/Egypt/N17040A/2019	NST	NGT	NVT	DTA	TDT	DRT	NST	---	---	N	H	T	A	N	L	I	G	T	N	P	D	N	L	N	T	T
A/chicken/Egypt/N17040B/2019	NST	NGT	NVT	DTA	TDT	DRT	NST	NIS	NGT	N	H	T	A	N	L	I	G	T	N	P	D	N	L	N	T	T
A/chicken/Egypt/N17041A/2019	---	---	---	---	---	---	---	---	NGT	-	-	-	-	-	-	-	-	-	-	-	-	-	-	-	-	-
A/chicken/Egypt/N17041C/2019	---	---	---	---	---	---	---	---	NGT	-	-	-	-	-	-	-	-	-	-	-	-	-	-	-	-	-
A/chicken/Egypt/N17048C/2019	NST	NGT	NVT	NTA	TDT	DRT	NST	NIS	NGT	N	H	T	A	N	L	I	G	T	N	P	D	N	L	N	T	T
A/chicken/Egypt/N17056/2019	NST	NGT	NVT	DTA	TDT	DRT	NST	NIS	NGT	N	H	T	A	N	L	I	G	T	N	P	D	N	L	N	T	T
A/chicken/Egypt/F17123A/2019	NST	NGT	NVT	DTA	TDT	DRT	NST	NIS	NGT	N	H	T	A	N	L	I	G	T	N	P	D	N	L	N	T	T
A/chicken/Egypt/F17124B/2019	NST	NGT	NVT	DTA	TDT	DRT	NST	NIS	NGT	N	H	T	A	N	L	I	G	T	N	P	D	N	L	N	T	T
A/chicken/Egypt/F17124C/2019	---	---	---	---	---	---	NST	NIS	NGT	-	-	-	-	-	-	-	-	-	-	-	-	-	-	-	-	-
A/chicken/Egypt/F17124D/2019	---	---	---	---	---	---	---	---	NGT	-	-	-	-	-	-	-	-	-	-	-	-	-	-	-	-	-
A/chicken/Egypt/F17133A/2019	---	---	---	---	---	---	---	---	NGT	-	-	-	-	-	-	-	-	-	-	-	-	-	-	-	-	-
A/chicken/Egypt/A17349/2019	NST	NGT	NVT	DTA	TDT	DRT	NST	NIS	NGT	N	H	T	A	N	L	I	G	T	N	P	D	N	L	N	T	T
A/chicken/Egypt/A17358/2019	NST	NGT	NVT	DTA	TDT	DRT	NRT	NIS	NGT	N	H	T	A	N	L	I	G	T	N	P	D	N	L	N	T	T
A/chicken/Egypt/A17416/2019	NST	NGT	NVT	DTT	TDT	DRT	NST	NIS	NGT	N	H	T	T	N	L	I	G	T	N	P	D	N	L	N	T	T
A/chicken/Egypt/A17561/2019	NST	NGT	NVT	DTA	TDT	DRT	NST	NIS	NGT	N	H	T	A	N	L	I	G	T	N	P	D	N	L	N	T	T
A/chicken/Egypt/S16694/2019	NST	NGT	NVT	DTA	TDT	DRT	NST	NIS	NGT	N	H	T	A	N	L	I	G	T	N	P	D	N	L	N	T	T
A/chicken/Egypt/S16688C/2019	NST	NGT	NVT	DTA	TDT	DRT	NST	NIS	NGT	N	H	T	A	N	L	I	G	T	N	P	D	N	L	N	T	T
A/chicken/Egypt/S16692/2019	NST	NGT	NVT	DTA	TDT	DRT	NST	NIS	NGT	N	H	T	A	N	L	I	G	T	N	P	D	N	L	N	T	T
A/chicken/Egypt/S16693/2019	NST	NGT	NVT	DTA	TDT	DRT	NST	NIS	NGT	N	H	T	A	N	L	I	G	T	N	P	D	N	L	N	T	T
A/chicken/Egypt/S16695/2019	NST	NGT	NVT	DTA	TDT	DRT	NST	NIS	NGT	N	H	T	A	N	L	I	G	T	N	P	D	N	L	N	T	T
A/chicken/Egypt/S16700B/2019	NST	NGT	NVT	DTA	TDT	DRT	NST	NIS	NGT	N	H	T	A	N	L	I	G	T	N	P	D	N	L	N	T	T
A/chicken/Egypt/S16700D/2019	NST	NGT	NVT	DTA	TDT	DRT	NST	NIS	NGT	N	H	T	A	N	L	I	G	T	N	P	D	N	L	N	T	T
A/chicken/Egypt/S16701B/2019	NST	NGT	NVT	DTA	TDT	DRT	NST	NIS	NGT	N	H	T	A	N	L	I	G	T	N	P	D	N	L	N	T	T
A/chicken/Egypt/D17949B/2019	NST	NGT	NVT	DTV	TDT	DRT	NST	NIS	NGT	N	H	T	V	N	L	I	G	T	N	P	D	N	L	N	T	T
A/chicken/Egypt/A17969/2019	NST	NGT	NVT	DTA	TDT	DRT	NST	NIS	NGT	N	H	T	A	N	L	I	G	T	N	P	Y	N	L	N	T	T

A/chicken/Egypt/D17949A/2019	NST	NGT	NVT	DTA	TDT	DRT	NST	NIS	NGT	N	H	T	A	N	L	I	G	T	N	P	D	N	L	N	T	T
A/chicken/Egypt/D17949C/2019	NST	NGT	NVT	DTA	TDT	DRT	NST	NIS	NGT	N	H	T	A	N	L	I	G	T	N	P	D	N	L	N	T	T
A/chicken/Egypt/Q17812B/2019	NST	NGT	NVT	DTA	TDT	DRT	NST	NIS	NGT	N	H	T	A	N	L	I	G	T	N	P	D	N	L	N	T	T
A/chicken/Egypt/Q17897C/2019	NST	NGT	NVT	DTA	TDT	DRT	NST	NIS	NGT	N	H	T	A	N	L	I	G	T	N	P	D	N	L	N	T	T
A/chicken/Egypt/Q18036B/2019	NST	NGT	NVT	DTA	TDT	DRT	NST	NIS	NGT	N	H	T	A	N	L	I	G	T	N	P	D	N	L	N	T	T
A/chicken/Egypt/Q18036E/2019	NST	NGT	NVT	DTA	TDT	DRT	NST	NIS	NGT	N	H	T	A	N	L	I	G	T	N	P	D	N	L	N	T	T
A/chicken/Egypt/S18034E/2019	NST	NGT	NVT	DTA	TDT	DRT	NST	NIS	NGT	N	H	T	A	N	L	I	G	T	N	P	D	N	L	N	T	T
A/pigeon/Egypt/A18092/2019	NST	NGT	NVT	DTA	TDT	DRT	NST	NIS	NGT	N	H	T	A	N	L	I	G	T	N	P	D	N	L	N	T	T
A/chicken/Egypt/Q18041A/2019	NST	NGT	NVT	DTA	TDT	DRA	NST	NIS	NGT	N	H	T	A	N	L	I	G	T	N	P	D	N	L	N	T	T
A/pigeon/Egypt/A18091/2019	NST	NGT	NVT	DTA	TDT	DRT	NST	NIS	NGT	N	H	T	A	N	L	I	G	T	N	P	D	N	L	N	T	T
A/pigeon/Egypt/A18093/2019	NST	NGT	NVT	DTA	TDT	DRN	NST	NIS	NGT	N	H	T	A	N	L	I	G	T	N	P	D	N	L	N	T	T
A/pigeon/Egypt/A18094/2019	NST	NGT	NVT	DTA	TDT	DRN	NST	NIS	NGT	N	H	T	A	N	L	I	G	T	N	P	D	N	L	N	T	T
A/chicken/Egypt/S16703B/2019	NST	NGT	NVT	DTA	TDT	DRT	NST	NIS	NGT	N	H	T	A	N	L	I	G	T	N	P	D	N	L	N	T	T
A/chicken/Egypt/A18717/2020	NST	NGT	NVT	DTA	TDT	DRT	NST	NIS	NGT	N	H	T	A	N	L	I	G	T	N	P	D	N	L	N	T	T
A/chicken/Egypt/A18720/2020	NST	NGT	NVT	DTA	TDT	DRT	NST	NIS	NGT	N	H	T	A	N	L	I	G	T	N	P	D	N	L	N	T	T
A/chicken/Egypt/A18716/2020	NST	NGT	NVT	DTA	TDT	DRT	NST	NIS	NGT	N	H	T	A	N	L	I	G	T	N	P	D	N	L	N	T	T
A/chicken/Egypt/S18643D/2020	NST	NGT	NVT	DTA	TDT	DRT	NST	NIS	NGT	N	H	T	A	N	L	I	G	T	N	P	D	N	L	N	T	T
A/chicken/Egypt/S18643C/2020	NST	NGT	NVT	DTA	TDT	DRT	NST	NIS	NGT	N	H	T	A	N	L	I	G	T	N	P	D	N	L	N	T	T
A/chicken/Egypt/A18610/2020	NST	NGT	NVT	DTV	TDT	DRT	NST	NIS	NGT	N	H	T	V	N	L	I	G	T	N	P	D	N	L	N	T	T
A/chicken/Egypt/A18607/2020	NST	NGT	NVT	DTT	TDT	DRT	NST	NIS	NGT	N	H	T	T	N	L	I	G	T	N	P	D	N	L	N	T	T
A/chicken/Egypt/A18605/2020	NST	NGT	NVT	DTA	TDT	DRT	NST	NIS	NGT	N	H	T	A	N	L	I	G	T	N	P	D	N	L	N	T	T
A/chicken/Egypt/A18505/2020	NST	NGT	NVT	DTA	TDT	DRT	NST	NIS	NGT	N	H	T	A	N	L	I	G	T	N	P	D	N	L	N	T	T
A/chicken/Egypt/A18507/2020	NST	NGT	NVT	DTA	TDT	DRT	NST	NIS	NGT	N	H	T	A	N	L	I	G	T	N	P	D	N	L	N	T	T
A/chicken/Egypt/D18579A/2020	NST	NGT	NVT	DTA	TDT	DRT	NST	NIS	NGT	N	H	T	A	N	L	I	G	T	N	P	D	N	L	N	T	T
A/chicken/Egypt/D18592/2020	NST	NGT	NVT	DTA	TDT	DRT	NST	NIS	NGT	N	H	T	A	N	L	I	G	T	N	P	D	N	L	N	T	T
A/chicken/Egypt/S18523C/2020	NST	NGT	NVT	DTA	TDT	DRT	NST	NIS	NGT	N	H	T	A	N	L	I	G	T	N	P	D	N	L	N	T	T
A/chicken/Egypt/S18527/2020	NST	NGT	NVT	DTA	TDT	DRT	NST	NIS	NGT	N	H	T	A	N	L	I	G	T	N	P	D	N	L	N	T	T
A/chicken/Egypt/A18495/2020	NST	NGT	NVT	DTA	TDT	DRT	NST	NIS	NGT	N	H	T	A	N	L	I	G	T	N	P	G	N	L	N	T	T
A/chicken/Egypt/A18503/2020	NST	NGT	NVT	DTA	TDT	DRT	NST	NIS	NGT	N	H	T	A	N	L	I	G	T	N	P	D	N	L	N	T	T

A/chicken/Egypt/A18504/2020	NST	NGT	NVT	DTA	TDT	DRT	NST	NIS	NGT	N	H	T	A	N	L	I	G	T	N	P	D	N	L	N	T	T
A/chicken/Egypt/A18496/2020	NST	NGT	NVT	DTA	TDT	DRT	NST	NIS	NGT	N	H	T	A	N	L	I	G	T	N	P	G	N	L	N	T	T
A/chicken/Egypt/A18498/2020	NST	NGT	NVT	DTA	TDT	DRT	NST	NIS	NGT	N	H	T	A	N	L	I	G	T	N	P	G	N	L	N	T	T
A/chicken/Egypt/F18299B/2020	NST	NGT	NVT	DTA	TDT	DRT	NST	NIS	NGT	N	H	T	A	N	L	I	G	T	N	P	D	N	L	N	T	T
A/chicken/Egypt/A18497/2020	NST	NGT	NVT	DTA	TDT	DRT	NST	NIS	NGT	N	H	T	A	N	L	I	G	T	N	P	G	N	L	N	T	T
A/chicken/Egypt/F18299C/2020	NST	NGT	NVT	DTA	TDT	DRT	NST	NIS	NGT	N	H	T	A	N	L	I	G	T	N	P	D	N	L	N	T	T
A/Chicken/Egypt/A19244/2020	NST	NGT	NVT	DTA	TDT	DRT	NST	NIS	NGT	N	H	T	A	N	L	I	G	T	N	P	D	N	L	N	T	T
A/Chicken/Egypt/A19245/2020	NST	NGT	NVT	DTA	TDT	DRT	NST	NIS	NGT	N	H	T	A	N	L	I	G	T	N	P	D	N	L	N	T	T
A/Chicken/Egypt/A19246/2020	NST	NGT	NVT	DTA	TDT	DRT	NST	NIS	NGT	N	H	T	A	N	L	I	G	T	N	P	D	N	L	N	T	T
A/Chicken/Egypt/D19290A/2020	NST	NGT	NVT	DTA	TDT	DRT	NST	NIS	NGT	N	H	T	A	N	L	I	G	T	N	P	D	N	L	N	T	T
A/Chicken/Egypt/D19290D/2020	NST	NGT	NVT	DTA	TDT	DRT	NST	NIS	NGT	N	H	T	A	N	L	I	G	T	N	P	D	N	L	N	T	T
A/Chicken/Egypt/D19293/2020	NST	NGT	NVT	DTA	TDT	DRT	NST	NIS	NGT	N	H	T	A	N	L	I	G	T	N	P	D	N	L	N	T	T
A/Chicken/Egypt/N18851C/2020	NST	NGT	NVT	DTA	TDT	DRT	NST	NIS	NGT	N	H	T	A	N	L	I	G	T	N	P	D	N	L	N	T	T
A/Chicken/Egypt/N19072B/2020	NST	NGT	NVT	DTA	TDT	DRT	NST	NIS	NGT	N	H	T	A	N	L	I	G	T	N	P	D	N	L	N	T	T
A/Chicken/Egypt/N19072C/2020	NST	NGT	NVT	DTA	TDT	DRT	NST	NIS	NGT	N	H	T	A	N	L	I	G	T	N	P	D	N	L	N	T	T
A/Chicken/Egypt/N19078B/2020	NST	NGT	NVT	DTA	TDT	DRT	NST	NIS	NGT	N	H	T	A	N	L	I	G	T	N	P	D	N	L	N	T	T
A/Chicken/Egypt/S18985A/2020	NST	NGT	NVT	DTA	TDT	DRT	NST	NIS	NGT	N	H	T	A	N	L	I	G	T	N	P	N	N	L	N	T	T
A/Chicken/Egypt/S18985B/2020	NST	NGT	NVT	DTA	TDT	DRT	NST	NIS	NGT	N	H	T	A	N	L	I	G	T	N	P	N	N	L	N	T	T
A/Chicken/Egypt/S18992/2020	NST	NGT	NVT	DTA	TDT	DRT	NST	NIS	NGT	N	H	T	A	N	L	I	G	T	N	P	N	N	L	N	T	T
A/Chicken/Egypt/S19326A/2020	NST	NGI	NVT	DTA	TDT	DRT	NST	NIS	NGT	N	H	T	A	N	L	I	G	T	N	P	D	N	L	N	T	T
A/Chicken/Egypt/S19326B/2020	NST	NGI	NVT	DTA	TDT	DRT	NST	NIS	NGT	N	H	T	A	N	L	I	G	T	N	P	D	N	L	N	T	T
A/Chicken/Egypt/S19326C/2020	NST	NGT	NVT	DTA	TDT	DRT	NST	NIS	NGT	N	H	T	A	N	L	I	G	T	N	P	D	N	L	N	T	T
A/Chicken/Egypt/S19326D/2020	NST	NGT	NVT	DTA	TDT	DRT	NST	NIS	NGT	N	H	T	A	N	L	I	G	T	N	P	D	N	L	N	T	T
A/Chicken/Egypt/S19338A/2020	NST	NGT	NVT	DTA	TDT	DRT	NST	NIS	NGT	N	H	T	A	N	L	I	G	T	N	P	D	N	L	N	T	T
A/Chicken/Egypt/S19338B/2020	NST	NGT	NVT	DTA	TDT	DRT	NST	NIS	NGT	N	H	T	A	N	L	I	G	T	N	P	D	N	L	N	T	T
A/Chicken/Egypt/S19338D/2020	NST	NGT	NVT	DTT	TDT	DRT	NST	NIS	NGT	N	H	T	T	N	L	I	G	T	N	P	D	N	L	N	T	T
A/Chicken/Egypt/A19165/2020	NST	NGT	NVT	DTA	TDT	DRT	NST	NIS	NGT	N	H	T	A	N	L	I	G	T	N	P	D	N	L	N	T	T
A/Duck/Egypt/D19288B/2020	NST	NGT	NVT	DTA	TDT	DRT	NST	NIS	NGT	N	H	T	A	N	L	I	G	T	N	P	D	N	L	N	T	T
A/Chicken/Egypt/D19290B/2020	---	---	---	---	---	---	NST	NIS	NGT	-	-	-	-	-	-	-	-	-	-	-	-	-	-	-	-	-
A/Chicken/Egypt/D19290C/2020	NST	NGT	NVT	DTA	TDT	DRT	NST	NIS	NGT	N	H	T	A	N	L	I	G	T	N	P	D	N	L	N	T	T
A/Chicken/Egypt/D19292/2020	NST	NGT	NVT	DTA	TDT	DRT	NST	NIS	NGT	N	H	T	A	N	L	I	G	T	N	P	D	N	L	N	T	T

A/Chicken/Egypt/N19074A/2020	NST	NGT	NVT	DTA	TDT	DRT	NST	NIS	NGT	N	H	T	A	N	L	I	G	T	N	P	D	N	L	N	T	T
A/Chicken/Egypt/N19302B/2020	NST	NGT	NVT	DTA	TDT	DRN	NST	NIS	NGT	N	H	T	A	N	L	I	G	T	N	P	D	N	L	N	T	T
A/Chicken/Egypt/N19304A/2020	NST	NGT	NVT	DTA	TDT	DRN	NST	NIS	NGT	N	H	T	A	N	L	I	G	T	N	P	D	N	L	N	T	T
A/Chicken/Egypt/S18755A/2020	NST	NGT	NVT	DTA	TDT	DRT	NST	NIS	NGT	N	H	T	A	N	L	I	G	T	N	P	D	N	L	N	T	T
A/Chicken/Egypt/S18755B/2020	NST	NGT	NVT	DTA	TDT	DRT	NST	NIS	NGT	N	H	T	A	N	L	I	G	T	N	P	D	N	L	N	T	T
A/Chicken/Egypt/S18755C/2020	NST	NGT	NVT	DTA	TDT	DRT	NST	NIS	NGT	N	H	T	A	N	L	I	G	T	N	P	D	N	L	N	T	T
A/Chicken/Egypt/S18755D/2020	---	---	---	DTA	TDT	DRT	NST	NIS	---	-	H	T	A	N	L	I	G	-	-	-	-	N	L	-	T	T
A/Chicken/Egypt/A19610/2021	NST	NGT	NVT	DTA	TDT	DRT	NST	NIS	NGT	N	H	T	A	N	L	I	G	T	N	P	D	N	L	N	T	T
A/Chicken/Egypt/A19618/2021	NST	NGT	NVT	DTA	TDT	DRI	NST	NIS	NGT	N	H	T	A	N	L	I	G	T	N	P	D	N	L	N	T	T
A/Chicken/Egypt/A19669/2021	NST	NGT	NVT	DTA	TDT	DRT	NST	NIS	NGT	N	H	T	A	N	L	I	G	T	N	P	D	N	L	N	T	T
A/Pigeon/Egypt/D19471/2020	NST	NGT	NVT	DTA	TDT	DRT	NST	NIS	NGT	N	H	T	A	N	L	I	G	T	N	P	D	N	L	N	T	T
A/Chicken/Egypt/D19479/2020	NST	NGT	NVT	DTA	TDT	DRT	NST	NIS	NGT	N	H	T	A	N	L	I	G	T	N	P	D	N	L	N	T	T
A/Chicken/Egypt/D19481/2020	NST	NGT	NVT	DTA	TDT	DRT	NST	NIS	NGT	N	H	T	A	N	L	I	G	T	N	P	D	N	L	N	T	T
A/Chicken/Egypt/D19486A/2020	NST	NGT	NVT	DTA	TDT	DRT	NST	NIS	NGT	N	H	T	A	N	L	I	G	T	N	P	D	N	L	N	T	T
A/Chicken/Egypt/D19486B/2020	NST	NGT	NVT	DTA	TDT	DRT	NST	NIS	NGT	N	H	T	A	N	L	I	G	T	N	P	D	N	L	N	T	T
A/Chicken/Egypt/N19598A/2021	NST	NGT	NVT	DTA	TDT	DRT	NST	NIS	NGT	N	H	T	A	N	L	I	G	T	N	P	D	N	L	N	T	T
A/Chicken/Egypt/N19598C/2021	NST	NGT	NVT	DTA	TDT	DRT	NST	NIS	NGT	N	H	T	A	N	L	I	G	T	N	P	D	N	L	N	T	T
A/Chicken/Egypt/N19598D/2021	NST	NGT	NVT	DTA	TDT	DRT	NST	NIS	NGT	N	H	T	A	N	L	I	G	T	N	P	D	N	L	N	T	T
A/Chicken/Egypt/N19599A/2021	NST	NGT	NVT	DTA	TDT	DRT	NST	NIS	NGT	N	H	T	A	N	L	I	G	T	N	P	D	N	L	N	T	T
A/Chicken/Egypt/N19600A/2021	NST	NGT	NVT	DTA	TDT	DRT	NST	NIS	NGT	N	H	T	A	N	L	I	G	T	N	P	D	N	L	N	T	T
A/Chicken/Egypt/N19600C/2021	NST	NGT	NVT	DTA	TDT	DRT	NST	NIS	NGT	N	H	T	A	N	L	I	G	T	N	P	D	N	L	N	T	T
A/Chicken/Egypt/N19600D/2021	NST	NGT	NVT	DTA	TDT	DRT	NST	NIS	NGT	N	H	T	A	N	L	I	G	T	N	P	D	N	L	N	T	T
A/Chicken/Egypt/N19604C/2021	NST	NGT	NVT	DTV	TDT	DRT	NST	NIS	NGT	N	H	T	V	N	L	I	G	T	N	P	D	N	L	N	T	T
A/Chicken/Egypt/N19604D/2021	NST	NGT	NVT	DTA	TDT	DRT	NST	NIS	NGT	N	H	T	A	N	L	I	G	T	N	P	D	N	L	N	T	T
A/Chicken/Egypt/N19606C/2021	NST	NGT	NVT	DTA	TDT	DRT	NST	NIS	NGT	N	H	T	A	N	L	I	G	T	N	P	D	N	L	N	T	T
A/Chicken/Egypt/N19764B/2021	NST	NGT	NVT	DTA	TDT	DRT	NST	NIS	NGT	N	H	T	A	N	L	I	G	T	N	P	D	N	L	N	T	T
A/Chicken/Egypt/N19764C/2021	NST	NGT	NVT	DTA	TDT	DRT	NST	NIS	NGT	N	H	T	A	N	L	I	G	T	N	P	D	N	L	N	T	T
A/Chicken/Egypt/N19766D/2021	NST	NGT	NVT	DTA	TDT	DRT	NST	NIS	NGT	N	H	T	A	N	L	I	G	T	N	P	D	N	L	N	T	T
A/Chicken/Egypt/S19708B/2021	NST	NGT	NVT	DTA	TDT	DRT	NST	NIS	NGT	N	H	T	A	N	L	I	G	T	N	P	D	N	L	N	T	T
A/Chicken/Egypt/S19712/2021	NST	NGT	NVT	DTA	TDT	DRT	NST	NIS	NGT	N	H	T	A	N	L	I	G	T	N	P	D	N	L	N	T	T

A/Chicken/Egypt/S19714/2021	NST	NGT	NVT	DTA	TDT	DRT	NST	NIS	NGT	N	H	T	A	N	L	I	G	T	N	P	D	N	L	N	T	T

Table S5. Comparison of the glycosylation sites of the NA of H9N2 viruses isolated from poultry in Egypt between 2017-2021.

**H9N2 Viruses**	**Glycosylation Sites**								
	44	61	69	86	146	200	234	298	402
A/chicken/Egypt/A14733/2017	NTS	NIT	NGT	NWS	NGT	NAT	NGT	SNR	NWS
A/pigeon/Egypt/D14794/2017	NTS	NIT	NGT	NWS	NGT	NAT	NGT	SNR	SWS
A/chicken/Egypt/F14014E/2017	NTS	NIT	NGT	NWS	NGT	NAT	NGT	SNR	NWS
A/chicken/Egypt/N13851C/2017	NTS	NIT	NGT	NWS	NGT	NAT	NGT	SNR	DWS
A/chicken/Egypt/N14498A/2017	NTS	NIT	NGT	NWS	NGT	NAT	NGT	SNR	NWS
A/chicken/Egypt/N14499B/2017	NTS	NIT	NGT	NWS	NGT	NAT	NGT	SNR	NWS
A/chicken/Egypt/N14500A/2017	NTS	NIT	NGT	NWS	NGT	NAT	NGT	SNR	NWS
A/chicken/Egypt/N14782D/2017	NTS	NIT	NGT	NWS	NGT	NAT	NGT	SNR	SWS
A/chicken/Egypt/Q14260A/2017	NTS	NIT	NGT	NWS	NGT	NAT	NGT	SNR	NWS
A/chicken/Egypt/S14165/2017	NTS	NIT	NGT	NWS	NGT	NAT	NGT	SNR	NWS
A/chicken/Egypt/S14187B/2017	NTS	NIT	NGT	SWS	NGT	NAT	NGT	SNR	NWS
A chicken/Egypt/A15043/2018	NTS	NIT	NGT	NWS	NGT	NAT	NGT	SNR	NWS
A chicken/Egypt/A15066/2018	NTS	NIT	NGT	NWS	NGT	NAT	NGT	SNR	NWS
A chicken/Egypt/A15068/2018	NTS	NIT	NGT	NWS	NGT	NAT	NGT	SNR	NWS
A chicken/Egypt/A15073/2018	NTS	NIT	NGT	NWS	NGT	NAT	NGT	SNR	DWS
A/CHICKEN/EGYPT/A15074/2018	NTS	NIT	NGT	NWS	NGT	NAT	NGT	SNR	DWS
A/CHICKEN/EGYPT/A15076/2018	NTS	NIT	NGT	NWS	NGT	NAT	NGT	SNR	DWS
A/CHICKEN/EGYPT/A15333/2018	NTS	NIT	NGT	NWS	NDT	NAT	NGT	SNR	NWS
A/CHICKEN/EGYPT/A15553C/2018	NTS	NIT	NGT	NWS	NGT	NAT	NGT	SNR	NWS
A/CHICKEN/EGYPT/A15573/2018	NTS	NIT	NGT	NWS	NGT	NAT	NGT	SNR	NWS
A/CHICKEN/EGYPT/A15582/2018	NTS	NIT	NGT	NWS	NGT	NAT	NGT	SNR	NWS
A/CHICKEN/EGYPT/A15659/2018	NTS	NIT	NGT	NWS	NGT	NAT	NGT	SNR	NWS
A/CHICKEN/EGYPT/A15660/2018	NTS	NIT	NGT	NWS	NGT	NAT	NGT	SNR	NWS
A/CHICKEN/EGYPT/A15669/2018	NTS	NIT	NGT	NWS	NGT	NAT	NGT	SNR	NWS
A/CHICKEN/EGYPT/A15799/2018	NTS	NIT	NGT	NWS	NGT	NAT	NGT	SNR	NWS
A/CHICKEN/EGYPT/A15801/2018	NTS	NIT	NGT	NWS	NGT	NAT	NGT	SNR	NWS
A/CHICKEN/EGYPT/A15802/2018	NTS	NIT	NGT	NWS	NGT	NAT	NGT	SNR	NWS
A/CHICKEN/EGYPT/A15803/2018	NTS	NIT	NGT	NWS	NGT	NAT	NGT	SNR	NWS
A/CHICKEN/EGYPT/D15830D/2018	NTS	NIT	NGT	NWS	NGT	NAT	NGT	SNR	SWS
A/CHICKEN/EGYPT/N15888/2018	NTS	NIT	NGN	NWS	NGT	NAT	NGT	SNR	NWS
A/CHICKEN/EGYPT/S15516/2018	NTS	NIT	NGT	NWS	NGT	NAT	NGT	SNR	NWS
A/CHICKEN/EGYPT/S15518/2018	NTS	NIT	NGT	NWS	NGT	NAT	NGT	SNR	NWS
A/CHICKEN/EGYPT/S15530C/2018	NTS	NIT	NGT	NWS	NGT	NAT	NGT	SNR	NWS
A/chicken/Egypt/S16702D/2019	NTS	NIT	NGT	NWS	NGT	NAT	NGT	SNR	SWS
A/chicken/Egypt/S16703C/2019	NTS	NIT	NGT	NWS	NGT	NAT	NGT	SNR	SWS
A/chicken/Egypt/S16703D/2019	NTS	NIT	NGT	NWS	NGT	NAT	NGT	SNR	SWS
A/chicken/Egypt/S16704A/2019	NTS	NIT	NGT	NWS	NGT	NAT	NGT	SNR	SWS
A/chicken/Egypt/S16704B/2019	NTS	NIT	NGT	NWS	NGT	NAT	NGT	SNR	SWS
A/chicken/Egypt/S16704C/2019	NTS	NIT	NGT	NWS	NGT	NAT	NGT	SNR	SWS
A/chicken/Egypt/S16705A/2019	NTS	NIT	NGT	NWS	NGT	NAT	NGT	SNR	SWS
A/chicken/Egypt/S16705C/2019	NTS	NIT	NGT	NWS	NGT	NAT	NGT	SNR	SWS
A/chicken/Egypt/S16705D/2019	NTS	NIT	NGT	NWS	NGT	NAT	NGT	SNR	SWS
A/chicken/Egypt/N16735D/2019	NTS	NIT	NGT	NWS	NGT	NAT	NGT	SNR	NWS
A/chicken/Egypt/N16739A/2019	NTS	NIT	NGT	NWS	NGT	NAT	NGT	SNR	NWS
A/chicken/Egypt/F16772D/2019	NTS	NIT	NGT	NWS	NGT	NAT	NGT	SNR	NWS
A/chicken/Egypt/F16774A/2019	NTS	NIT	NGT	NWS	NGT	NAT	NGT	SNR	NWS
A/chicken/Egypt/F16774B/2019	NTS	NIT	NGT	NWS	NGT	NAT	NGT	SNR	NWS
A/chicken/Egypt/A16777/2019	NIS	NIT	NGT	NWS	NGT	NAT	NGT	SNR	NWS
A/chicken/Egypt/A16857/2019	NTS	NIT	NGT	NWS	NGT	NAT	NGT	SNR	NWS
A/chicken/Egypt/A16859/2019	NTS	NIT	NGT	NWS	NGT	NAT	NGT	SNR	NWS
A/chicken/Egypt/A16860/2019	NTS	NIT	NGT	NWS	NGT	NAT	NGT	SNR	NWS
A/chicken/Egypt/A16863/2019	NTS	NIT	NGT	NWS	NGT	NAT	NGT	SNR	NWS
A/pigeon/Egypt/A16865/2019	NTS	NIT	NGT	NWS	NGT	NAT	NGT	SNR	NWS

A/chicken/Egypt/A16886/2019	NTS	NIT	NGT	NWS	NGT	NAT	NGT	SNR	NWS
A/chicken/Egypt/N16941C/2019	NTS	NIT	NGT	NWS	NGT	NAT	NGT	SNR	NRS
A/chicken/Egypt/N16946C/2019	NTS	NIT	NGT	NWS	NGT	NAT	NGT	SNR	NRS
A/chicken/Egypt/S16965D/2019	NTS	NIT	NGT	NWS	NGT	NAT	NGT	SNR	SWS
A/chicken/Egypt/N17040A/2019	NTS	NIT	NGT	NWS	NGT	NAT	NGT	SNR	NWS
A/chicken/Egypt/N17040B/2019	NTS	NIT	NGT	NWS	NGT	NAT	NGT	SNR	NWS
A/chicken/Egypt/N17041A/2019	NTS	NIT	NGT	NWS	NGT	NAT	NGT	SNR	NWS
A/chicken/Egypt/N17041C/2019	NTS	NIT	NGT	NWS	NGT	NAT	NGT	SNR	NWS
A/chicken/Egypt/N17048C/2019	NTS	NIT	NGT	NWS	NGT	NAT	NGT	SNR	NWS
A/chicken/Egypt/N17056/2019	NTS	NIT	NGT	NWS	NGT	NAT	NGT	SNR	NWS
A/chicken/Egypt/F17123A/2019	NTS	NIT	NGT	NWS	NGT	NAT	NGT	SNR	NWS
A/chicken/Egypt/F17124B/2019	NTS	NIT	NGT	NWS	NGT	NAT	NGT	SNR	NWS
A/chicken/Egypt/F17124C/2019	NTS	NIT	NGT	NWS	NGT	NAT	NGT	SNR	NWS
A/chicken/Egypt/F17124D/2019	NTS	NIT	NGT	NWS	NGT	NAT	NGT	SNR	NWS
A/chicken/Egypt/F17133A/2019	NTS	NIT	NGT	NWS	NGT	NAT	NGT	SNR	SWS
A/chicken/Egypt/A17349/2019	NTS	NIT	NGT	NWS	NGT	NAT	NGT	SNR	SWS
A/chicken/Egypt/A17358/2019	NTS	NIT	NGT	NWS	NGT	NAT	NGT	SNR	SWS
A/chicken/Egypt/A17416/2019	NTS	NIT	NGT	NWS	NGT	NAT	NGT	SNR	SWS
A/chicken/Egypt/A17561/2019	NTS	NIT	NGT	NWS	NGT	NAT	NGT	SNR	NWS
A/chicken/Egypt/S16694/2019	NTS	NIT	NGT	NWS	NGT	NAT	NGT	SNR	SWS
A/chicken/Egypt/S16688C/2019	NTS	NIT	NGT	NWS	NGT	NAT	NGT	SNR	SWS
A/chicken/Egypt/S16692/2019	NTS	NIT	NGT	NWS	NGT	NAT	NGT	SNR	SWS
A/chicken/Egypt/S16693/2019	NTS	NIT	NGT	NWS	NGT	NAT	NGT	SNR	SWS
A/chicken/Egypt/S16695/2019	NTS	NIT	NGT	NWS	NGT	NAT	NGT	SNR	SWS
A/chicken/Egypt/S16700B/2019	NTS	NIT	NGT	NWS	NGT	NAT	NGT	SNR	SWS
A/chicken/Egypt/S16700D/2019	NTS	NIT	NGT	NWS	NGT	NAT	NGT	SNR	SWS
A/chicken/Egypt/S16701B/2019	NTS	NIT	NGT	NWS	NGT	NAT	NGT	SNR	SWS
A/chicken/Egypt/D17949B/2019	NTS	NIT	NGT	NWS	NGT	NAT	NGT	SNR	NWS
A/chicken/Egypt/A17969/2019	NTS	NIT	NGT	SWS	NGT	NAT	NGT	SNR	NWS
A/chicken/Egypt/D17949A/2019	NTS	NIT	NGT	NWS	NGT	NAT	NGT	SNR	NWS
A/chicken/Egypt/D17949C/2019	NTS	NIT	NGT	NWS	NGT	NAT	NGT	SNR	NWS
A/chicken/Egypt/Q17812B/2019	NTS	NIT	NGT	NWS	NGT	NAT	NGT	SNR	NWS
A/chicken/Egypt/Q17897C/2019	NTS	NIT	NGT	NWS	NGT	NAT	NGT	SNR	NWS
A/chicken/Egypt/Q18036B/2019	NTS	NIT	NGT	NWS	NGT	NAT	NGT	SNR	NWS
A/chicken/Egypt/Q18036E/2019	NTS	NIT	NGT	NWS	NGT	NAT	NGT	SNR	NWS
A/chicken/Egypt/S18034E/2019	NTS	NIT	NGT	NWS	NGT	NAT	NGT	SNR	NWS
A/pigeon/Egypt/A18092/2019	NIS	NIT	NGT	NWS	NGT	NAT	NGT	SNR	NWS
A/chicken/Egypt/Q18041A/2019	NTS	NIT	NGT	NWS	NGT	NAT	NGT	SNR	NWS
A/pigeon/Egypt/A18091/2019	NTS	NIT	NGT	NWS	NGT	NAT	NGT	SNR	NWS
A/pigeon/Egypt/A18093/2019	NIS	NIT	NGT	NWS	NGT	NAT	NGT	SNR	NWS
A/pigeon/Egypt/A18094/2019	NTS	NIT	NGT	NWS	NGT	NAT	NGT	SNR	NWS
A/chicken/Egypt/S16703B/2019	NTS	NIT	NGT	NWS	NGT	NAT	NGT	SNR	SWS
A/chicken/Egypt/A18717/2020	NTS	SIT	NGT	NWS	NGT	NAT	DGT	SNR	NWS
A/chicken/Egypt/A18720/2020	NTS	SIT	NGT	NWS	NGT	NAT	DGT	SNR	NWS
A/chicken/Egypt/A18716/2020	NTS	SIT	NGT	NWS	NGT	NAT	DGT	SNR	NWS
A/chicken/Egypt/S18643D/2020	NTS	NIT	NGT	NWS	NGT	NAT	NGT	SNR	SWS
A/chicken/Egypt/S18643C/2020	NTS	NIT	NGT	NWS	NGT	NAT	NGT	SNR	SWS
A/chicken/Egypt/A18610/2020	NTS	NIT	NGT	NWS	NGT	NAT	NGT	SNR	NWS
A/chicken/Egypt/A18607/2020	NTS	NIT	NGT	NWS	NGT	NAT	NGT	SNR	NWS
A/chicken/Egypt/A18605/2020	NTS	NIT	NGT	NWS	NGT	NAT	NGT	SNR	NWS
A/chicken/Egypt/A18505/2020	NTS	NIT	NGT	NWS	NGT	NAT	NGT	SNR	NWS
A/chicken/Egypt/A18507/2020	NTS	NIT	NGT	NWS	NGT	NAT	NGT	SNR	NWS
A/chicken/Egypt/D18579A/2020	NTS	NIT	NGT	NWS	NGT	NAT	NGT	SNR	NWS
A/chicken/Egypt/D18592/2020	NTS	NIT	NGT	NWS	NGT	NAT	NGT	SNR	NWS
A/chicken/Egypt/S18523C/2020	NTS	NIT	NGT	NWS	NGT	NAT	NGT	SNR	NWS
A/chicken/Egypt/S18527/2020	NTS	NIT	NGT	NWS	NGT	NAT	NGT	SNR	NWS
A/chicken/Egypt/A18495/2020	NTS	NIT	NGT	NWS	NGT	NAT	NGT	SNR	NWS
A/chicken/Egypt/A18503/2020	NTS	NIT	NGT	NWS	NGT	NAT	NGT	SNR	NWS
A/chicken/Egypt/A18504/2020	NTS	NIT	NGT	NWS	NGT	NAT	NGT	SNR	NWS
A/chicken/Egypt/A18496/2020	NTS	NIT	NGT	NWS	NGT	NAT	NGT	SNR	NWS

A/chicken/Egypt/A18498/2020	NTS	NIT	NGT	NWS	NGT	NAT	NGT	SNR	NWS
A/chicken/Egypt/F18299B/2020	NTS	NIT	NGT	NWS	NGT	NAT	NGT	SNR	NRS
A/chicken/Egypt/A18497/2020	NTS	NIT	NGT	NWS	NGT	NAT	NGT	SNR	NWS
A/chicken/Egypt/F18299C/2020	NTS	NIT	NGT	NWS	NGT	NAT	NGT	SNR	NRS
A/Chicken/Egypt/A19244/2020	NTS	NIT	NGT	NWS	NGT	NAT	DGT	SNR	NWS
A/Chicken/Egypt/A19245/2020	NTS	NIT	NGT	NWS	NGT	NAT	DGT	SNR	NWS
A/Chicken/Egypt/A19246/2020	NTS	NIT	NGT	NWS	NGT	NAT	DGT	SNR	NWS
A/Chicken/Egypt/D19290A/2020	NTS	NIT	NGT	NWS	NGT	NAT	NGT	SNR	NWS
A/Chicken/Egypt/D19290D/2020	NTS	NIT	NGT	NWS	NGT	NAT	NGT	SNR	NWS
A/Chicken/Egypt/D19293/2020	NTS	NIT	NGT	NWS	NGT	NAT	NGT	SNR	NWS
A/Chicken/Egypt/N18851C/2020	NTS	NIT	NGT	NWS	NGT	NAT	DGT	SNR	NWS
A/Chicken/Egypt/N19072B/2020	NTS	NIT	NGT	NWS	NGT	NAT	DGT	SNR	NWS
A/Chicken/Egypt/N19072C/2020	NTS	NIT	NGT	NWS	NGT	NAT	DGT	SNR	NWS
A/Chicken/Egypt/N19078B/2020	NTS	NIT	NGT	NWS	NGT	NAT	DGT	SNR	NWS
A/Chicken/Egypt/S18985A/2020	NTS	NIT	NGT	NWS	NGT	NAT	NGT	SNR	NRS
A/Chicken/Egypt/S18985B/2020	NTS	NIT	NGT	NWS	NGT	NAT	NGT	SNR	NRS
A/Chicken/Egypt/S18992/2020	NTS	NIT	NGT	NWS	NGT	NAT	NGT	SNR	NRS
A/Chicken/Egypt/S19326A/2020	NTS	NIT	NGT	NWS	NGT	NAT	NGT	SNR	NWS
A/Chicken/Egypt/S19326B/2020	NTS	NIT	NGT	NWS	NGT	NAT	NGT	SNR	NWS
A/Chicken/Egypt/S19326C/2020	NTS	NIT	NGT	NWS	NGT	NAT	NGT	SNR	NWS
A/Chicken/Egypt/S19326D/2020	NTS	NIT	NGT	NWS	NGT	NAT	NGT	SNR	NWS
A/Chicken/Egypt/S19338A/2020	NTS	NIT	NGT	NWS	NGT	NAT	NGT	SNR	NWS
A/Chicken/Egypt/S19338B/2020	NTS	NIT	NGT	NWS	NGT	NAT	NGT	SNR	NWS
A/Chicken/Egypt/S19338D/2020	NTS	NIT	NGT	NWS	NGT	NAT	NGT	SNR	NWS
A/Chicken/Egypt/A19165/2020	NIS	NIT	NGT	NWS	NGT	NAT	NGT	SNR	NWS
A/Duck/Egypt/D19288B/2020	NTS	NIT	NGT	NWS	NGT	NAT	NGT	SNR	NWS
A/Chicken/Egypt/D19290B/2020	---	---	---	---	NGT	NAT	NGT	SNR	NWS
A/Chicken/Egypt/D19290C/2020	NTS	NIT	NGT	NWS	NGT	NAT	NGT	SNR	NWS
A/Chicken/Egypt/D19292/2020	NTS	NIT	NGT	NWS	NGT	NAT	NGT	SNR	NWS
A/Chicken/Egypt/N19074A/2020	---	---	---	---	---	NAT	DGT	SNR	NWS
A/Chicken/Egypt/N19302B/2020	NIS	NIT	NGT	NWS	NGT	NAT	NGT	SNR	NWS
A/Chicken/Egypt/N19304A/2020	NIS	NIT	NGT	NWS	NGT	NAT	NGT	SNR	NWS
A/Chicken/Egypt/S18755A/2020	TPQ	NIT	NGT	NWS	NGT	NAT	NGT	SNR	NWS
A/Chicken/Egypt/S18755B/2020	TPQ	NIT	NGT	NWS	NGT	NAT	NGT	SNR	NWS
A/Chicken/Egypt/S18755C/2020	TPQ	NIT	NGT	NWS	NGT	NAT	NGT	SNR	NWS
A/Chicken/Egypt/S18755D/2020	---	---	---	---	---	NAT	NGT	SNR	NWS
A/Chicken/Egypt/A19610/2021	NTS	NIT	NGT	NWS	NGT	NAT	DGT	SNR	NWS
A/Chicken/Egypt/A19618/2021	NTS	NIT	NGT	NWS	NGT	NAT	NGT	SNR	NRS
A/Chicken/Egypt/A19669/2021	NTS	NIT	NGT	NWS	NGT	NAT	NGT	SNR	NWS
A/Pigeon/Egypt/D19471/2020	NTS	NIT	NGT	NWS	NGT	NAT	NGT	SNR	NWS
A/Chicken/Egypt/D19479/2020	NTS	NIT	NGT	NWS	NGT	NAT	NGT	SNR	NWS
A/Chicken/Egypt/D19481/2020	NTS	NIT	NGT	NWS	NGT	NAT	NGT	SNR	NWS
A/Chicken/Egypt/D19486A/2020	NTS	NIT	NGT	NWS	NGT	NAT	NGT	SNR	NWS
A/Chicken/Egypt/D19486B/2020	NTS	NIT	NGT	NWS	NGT	NAT	NGT	SNR	NWS
A/Chicken/Egypt/N19598A/2021	NTS	NIT	NGT	NWS	NGT	NAT	NGT	SNR	NRS
A/Chicken/Egypt/N19598C/2021	NTS	NIT	NGT	NWS	NGT	NAT	NGT	SNR	NRS
A/Chicken/Egypt/N19598D/2021	NTS	NIT	NGT	NWS	NGT	NAT	NGT	SNR	NRS
A/Chicken/Egypt/N19599A/2021	NTS	NIT	NGT	NWS	NGT	NAT	NGT	SNR	NRS
A/Chicken/Egypt/N19600A/2021	NTS	NIT	NGT	NWS	NGT	NAT	NGT	SNR	NRS
A/Chicken/Egypt/N19600C/2021	NTS	NIT	NGT	NWS	NGT	NAT	NGT	SNR	NRS
A/Chicken/Egypt/N19600D/2021	NTS	NIT	NGT	NWS	NGT	NAT	NGT	SNR	NRS
A/Chicken/Egypt/N19604C/2021	NTS	NIT	NGT	NWS	NGT	NAT	NGT	SNR	NRS
A/Chicken/Egypt/N19604D/2021	NTS	NIT	NGT	NWS	NGT	NAT	NGT	SNR	NRS
A/Chicken/Egypt/N19606C/2021	NTS	NIT	NGT	NWS	NGT	NAT	NGT	SNR	NRS
A/Chicken/Egypt/N19764B/2021	NTS	NIT	NGT	NWS	NGT	NAT	NGT	SNR	NWS
A/Chicken/Egypt/N19764C/2021	NTS	NIT	NGT	NWS	NGT	NAT	NGT	SNR	NWS
A/Chicken/Egypt/N19766D/2021	NTS	NIT	NGT	NWS	NGT	NAT	NGT	SNR	NWS
A/Chicken/Egypt/S19708B/2021	NTS	NIT	NGT	NWS	NGT	NAT	NGT	SNR	NWS
A/Chicken/Egypt/S19712/2021	NTS	NIT	NGT	NWS	NGT	NAT	NGT	SNR	NWS
A/Chicken/Egypt/S19714/2021	NTS	NIT	NGT	NWS	NGT	NAT	NGT	SNR	NWS

Table S6. Titers of A/chicken/Egypt/S4456B/2011(H9N2) and A/chicken/Egypt/A17358/2019 (H9N2) viruses in organs obtained from infected chicken and duck groups. Data are the mean Log 10 EID50/ml ± SD for positive samples.

	dpi	Type of Organs	S4456B (Genotype I)	A17358 (Genotype III)
chickens	3	Trachea	<1 (0/3)*	6±2 (3/3)
		Lung	<1 (0/3)	3 (3/3)
		Spleen	<1 (0/3)	3 (1/3)
		Kidney	<1 (0/3)	2.6±1.5 (2/3)
		Liver	<1 (0/3)	<1 (0/3)
		Bursa	<1 (0/3)	2.1±1.2 (2/3)
		Intestine	<1 (0/3)	<1 (0/3)
		Brain	<1 (0/3)	<1 (0/3)
	6	Trachea	<1 (0/3)	<1 (0/3)
		Lung	<1 (0/3)	<1 (0/3)
		Spleen	<1 (0/3)	<1 (0/3)
		Kidney	<1 (0/3)	<1 (0/3)
		Liver	<1 (0/3)	<1 (0/3)
		Bursa	<1 (0/3)	<1 (0/3)
		Intestine	<1 (0/3)	<1 (0/3)
		Brain	<1 (0/3)	<1 (0/3)
Ducks	3	Trachea	<1 (0/3)	2 (1/3)
		Lung	<1 (0/3)	<1 (0/3)
		Spleen	<1 (0/3)	<1 (0/3)
		Kidney	<1 (0/3)	<1 (0/3)
		Liver	<1 (0/3)	<1 (0/3)
		Bursa	<1 (0/3)	<1 (0/3)
		Intestine	<1 (0/3)	<1 (0/3)
		Brain	<1 (0/3)	<1 (0/3)
	6	Trachea	<1 (0/3)	<1 (0/3)
		Lung	<1 (0/3)	<1 (0/3)
		Spleen	<1 (0/3)	<1 (0/3)
		Kidney	<1 (0/3)	<1 (0/3)
		Liver	<1 (0/3)	<1 (0/3)
		Bursa	<1 (0/3)	<1 (0/3)
		Intestine	<1 (0/3)	<1 (0/3)
		Brain	<1 (0/3)	<1 (0/3)

*Values in parentheses are number of positive chickens out of each group.

Table S7. Titers of A/chicken/Egypt/S4456B/2011(H9N2) and A/chicken/Egypt/A17358/2019 (H9N2) viruses in swabs obtained from infected chicken and duck groups. Data are the mean Log 10 EID50/ml ± SD for positive samples.

dpi	Type of Swabs	S4456B (Genotype I)	A17358 (Genotype III)
3	Oral chicken	2.3±0.5 (3/3)*	5.3±0.7 (3/3)
	Cloacal chicken	<1 (0/3)	3(1/3)
6	Oral chicken	<1 (0/5)	2.1(2/5)
	Cloacal chicken	<1 (0/5)	3.8±0.7 (3/5)
10	Oral chicken	<1 (0/5)	<1 (0/5)
	Cloacal chicken	<1 (0/5)	<1 (0/5)
3	Oral duck	3 (1/3)	2 (1/3)
	Cloacal duck	<1 (0/3)	<1 (0/3)
6	Oral duck	5.5±1(4/5)	2(1/5)
	Cloacal duck	6±0.0 (4/5)	3(1/5)
10	Oral duck	<1 (0/5)	<1 (0/5)
	Cloacal duck	<1 (0/5)	<1 (0/5)

*Values in parentheses are number of positive chickens out of each group.

References

1. Chen, G.-W.; Chang, S.-C.; Mok, C.-K.; Lo, Y.-L.; Kung, Y.-N.; Huang, J.-H.; Shih, Y.-H.; Wang, J.-Y.; Chiang, C.; Chen, C.-J. Genomic signatures of human versus avian influenza A viruses. *Emerging infectious diseases* **2006**, *12*, 1353.
2. Shaw, M.; Cooper, L.; Xu, X.; Thompson, W.; Krauss, S.; Guan, Y.; Zhou, N.; Klimov, A.; Cox, N.; Webster, R. Molecular changes associated with the transmission of avian influenza a H5N1 and H9N2 viruses to humans. *Journal of Medical Virology* **2002**, *66*, 107-114.
3. Li, J.; Li, Y.; Hu, Y.; Chang, G.; Sun, W.; Yang, Y.; Kang, X.; Wu, X.; Zhu, Q. PB1-mediated virulence attenuation of H5N1 influenza virus in mice is associated with PB2. *J Gen Virol* **2011**, *92*, 1435-1444, doi:10.1099/vir.0.030718-0.
4. Guilligay, D.; Tarendeau, F.; Resa-Infante, P.; Coloma, R.; Crepin, T.; Sehr, P.; Lewis, J.; Ruigrok, R.W.; Ortin, J.; Hart, D.J.; et al. The structural basis for cap binding by influenza virus polymerase subunit PB2. *Nat Struct Mol Biol* **2008**, *15*, 500-506, doi:10.1038/nsmb.1421.
5. Wang, J.; Sun, Y.; Xu, Q.; Tan, Y.; Pu, J.; Yang, H.; Brown, E.G.; Liu, J. Mouse-adapted H9N2 influenza A virus PB2 protein M147L and E627K mutations are critical for high virulence. *PloS one* **2012**, *7*, e40752.
6. Kuzuhara, T.; Kise, D.; Yoshida, H.; Horita, T.; Murazaki, Y.; Nishimura, A.; Echigo, N.; Utsunomiya, H.; Tsuge, H. Structural basis of the influenza A virus RNA polymerase PB2 RNA-binding domain containing the pathogenicity-determinant lysine 627 residue. *Journal of Biological Chemistry* **2009**, *284*, 6855-6860.
7. Zhou, B.; Pearce, M.B.; Li, Y.; Wang, J.; Mason, R.J.; Tumpey, T.M.; Wentworth, D.E. Asparagine substitution at PB2 residue 701 enhances the replication, pathogenicity, and transmission of the 2009 pandemic H1N1 influenza A virus. *PloS one* **2013**, *8*, e67616.
8. Taubenberger, J.K.; Reid, A.H.; Lourens, R.M.; Wang, R.; Jin, G.; Fanning, T.G. Characterization of the 1918 influenza virus polymerase genes. *Nature* **2005**, *437*, 889-893.
9. Wanitchang, A.; Jengarn, J.; Jongkaewwattana, A. The N terminus of PA polymerase of swine-origin influenza virus H1N1 determines its compatibility with PB2 and PB1 subunits through a strain-specific amino acid serine 186. *Virus research* **2011**, *155*, 325-333.
10. Wang, D.; Tang, G.; Huang, Y.; Yu, C.; Li, S.; Zhuang, L.; Fu, L.; Wang, S.; Li, N.; Li, X. A returning migrant worker with avian influenza A (H7N9) virus infection in Guizhou, China: a case report. *Journal of medical case reports* **2015**, *9*, 1-6.
11. Brown, E.; Liu, H.; Kit, L.C.; Baird, S.; Nesrallah, M. Pattern of mutation in the genome of influenza A virus on adaptation to increased virulence in the mouse lung: identification of functional themes. *Proceedings of the National Academy of Sciences* **2001**, *98*, 6883-6888.
12. Finkelstein, D.B.; Mukatira, S.; Mehta, P.K.; Obenauer, J.C.; Su, X.; Webster, R.G.; Naeve, C.W. Persistent host markers in pandemic and H5N1 influenza viruses. *Journal of virology* **2007**, *81*, 10292-10299.
13. Yamaji, R.; Yamada, S.; Le, M.Q.; Ito, M.; Sakai-Tagawa, Y.; Kawaoka, Y. Mammalian adaptive mutations of the PA protein of highly pathogenic avian H5N1 influenza virus. *Journal of virology* **2015**, *89*, 4117-4125.
14. Gabriel, G.; Dauber, B.; Wolff, T.; Planz, O.; Klenk, H.-D.; Stech, J. The viral polymerase mediates adaptation of an avian influenza virus to a mammalian host. *Proceedings of the National Academy of Sciences* **2005**, *102*, 18590-18595.
15. Katz, J.M.; Lu, X.; Tumpey, T.M.; Smith, C.B.; Shaw, M.W.; Subbarao, K. Molecular correlates of influenza A H5N1 virus pathogenesis in mice. *Journal of Virology* **2000**, *74*, 10807-10810.
16. Pan, C.; Jiang, S. E14-F55 combination in M2 protein: a putative molecular determinant responsible for swine-origin influenza A virus transmission in humans. *PLoS currents* **2009**, *1*.
17. Soubies, S.M.; Volmer, C.; Croville, G.; Loupias, J.; Peralta, B.; Costes, P.; Lacroux, C.; Guérin, J.-L.; Volmer, R. Species-specific contribution of the four C-terminal amino acids of influenza A virus NS1 protein to virulence. *Journal of virology* **2010**, *84*, 6733-6747.
